# Long-term effects of air pollution on daily outpatient visits for allergic conjunctivitis from 2013 to 2020: a time-series study in Urumqi, China

**DOI:** 10.3389/fpubh.2024.1325956

**Published:** 2024-10-25

**Authors:** Dongwei Liu, Siyu Gui, Xinchen Wang, Qianqian Wang, Jianchao Qiao, Fangbiao Tao, Liming Tao, Zhengxuan Jiang, Xianglong Yi

**Affiliations:** ^1^Department of Ophthalmology, The Second Affiliated Hospital of Anhui Medical University, Hefei, China; ^2^Department of Clinical Medicine, The Second School of Clinical Medicine, Anhui Medical University, Hefei, China; ^3^Department of Maternal, Child and Adolescent Health, School of Public Health, Anhui Medical University, Hefei, China; ^4^Department of Ophthalmology, The First Affiliated Hospital of Xinjiang Medical University, Ürümqi, China

**Keywords:** allergic conjunctivitis, air pollution, time-series analysis, outpatient visits, population

## Abstract

**Introduction:**

This study aimed to elucidate the effects of outdoor air pollution and allergic conjunctivitis and population-based lagged effects of air pollution.

**Methods:**

We included data on six major air pollutants, PM_10_, PM_2.5_, carbon monoxide (CO), sulfur dioxide (SO_2_), nitrogen dioxide (NO_2_), and ozone (O3), and 3325 allergic conjunctivitis outpatient visits in Urumqi, northwest China, from 1 January 2013 to 31 December 2020. We developed quasi-Poisson generalized linear regression models with distributed lagged nonlinear models (DLNM), and single and multi-pollutant models were constructed to investigate single-day and cumulative lagged effects in detail.

**Results:**

Our results confirmed that elevated PM_10_ and NO_2_ levels are significantly associated with increased allergic conjunctivitis outpatient visits with lags of 2 and 3 days respectively, and subgroup analyses further suggest that the effects of PM_10_ and NO_2_ on allergic conjunctivitis are more pronounced during the warm season. Women are more sensitive to PM_10_ exposure and the effect of air pollution on allergic conjunctivitis is influenced by age (e.g., infancy and older people).

**Discussion:**

Our work provides the first time-series study in Urumqi, the world's furthest inland city from the ocean. Further implementation of specific outdoor air pollution controls such as the burning of fossil fuels like coal, as well as special population protection policies remain necessary. Multicenter studies with larger sample sizes are needed.

## 1 Introduction

Urumqi is located in north-western China, which is the capital of the Xinjiang Uyghur Autonomous Region (hereinafter referred to as “Xinjiang”). Given its unique geographical location and the large temperature difference between day and night, the climate here is dry with even extreme weather such as sandstorms often occurring (1). Although Urumqi has a vast area of land, the actual habitable area is very small, so the population is exceptionally densely distributed. In terms of air quality, Urumqi is one of the most socially and economically developed cities in China, as well as one of the most rapidly urbanizing (2). With concerns about severe air pollution from industrialization, Urumqi is increasingly gaining attention as a typical case of a large Chinese city suffering from severe air pollution (3). Previously reported studies have demonstrated that the long-term annual average concentrations of PM_10_, SO_2_, and NO_2_ in the Urumqi region mostly exceed the Chinese CNAAQS secondary national standard, for example, annual average SO_2_ levels exceed the secondary standard (60 μg/m^3^), while annual average NO_2_ concentrations exceed their annual standard (40 μg/m^3^) by up to 1.3 times (4). The 2017 Global Burden of Disease Study also suggests that the region has the highest average PM_2.5_ levels and the highest age-standardized mortality rate per 100,000 people due to ambient particulate matter pollution compared to other regions in China (5). Considering the adverse effects of air pollution on human health due to urban construction and industrialization in recent years, such as metabolic diseases (6, 7), cardiovascular diseases (8, 9) and allergic diseases (10, 11) have been reported to be significantly associated with outdoor air pollution. However, few studies have focused on the relationship between ocular diseases and environmental pollution in the Urumqi region, let alone the development of allergic conjunctivitis that may be directly related to exposure to outdoor airborne allergens (12). To our knowledge, there are limited articles focusing on eastern as well as southern China (13, 14), and even less evidence of environmental epidemiological studies covering cities in western China, and given the ethnic differences, large population concentrations, and unique outdoor air pollution characteristics, it is necessary to clarify the exact effects of air pollution levels and types.

Allergic conjunctivitis is one of the common ocular surface diseases, which is characterized by redness, itching, burning sensation, photophobia, tearing, and mostly viscous ocular discharge of the conjunctival tissue, but the exact cause of the disease is still unknown (14). Allergic conjunctivitis can be divided into five main categories: seasonal allergic conjunctivitis (SAC), atopic keratoconjunctivitis (AKC), perennial allergic conjunctivitis (PAC), vernal keratoconjunctivitis (VKC) and giant papillary conjunctivitis (GPC) (15). Although the pathogenesis of allergic conjunctivitis is unknown, current research has confirmed that pollen, animal dander, and other environmental antigens may be the main cause of the development of allergic conjunctivitis (16). As a common cause of outpatient visits to ophthalmology clinics, the incidence of allergic conjunctivitis has been increasing worldwide. Allergic conjunctivitis has been reported to affect up to 40% of the US population (17). Similarly, with rapid industrialization and urbanization, the number of patients with allergic conjunctivitis in China has been increasing yearly, reaching 295 million in 2020 (1). However, even though allergic conjunctivitis will significantly reduce work and educational productivity and overall quality of life, with substantial negative effects on socioeconomic wellbeing, there is still a lack of detailed population-based studies, particularly on the effects of outdoor air pollution on allergic conjunctivitis. The limited number of previous studies on the effects of outdoor air pollution exposure on allergic conjunctivitis is inconsistent and may be related to several factors (11, 17). On the one hand, the ocular surface is directly exposed to the external atmosphere and is susceptible to airborne allergens such as pollen (18), dust, and air pollutants (8). On the other hand, however, the interaction of the cleaning system and the immune system of the ocular surface with different types of air pollutants may influence the penetration and retention of allergens, prolonging the exposure-response, and most previous studies have not considered the lagging effect of air pollutants, which may lead to inaccurate results. Therefore, it is essential to fully investigate the types of different air pollutants, the type of exposure (categorical exposure or continuous exposure), and the lag effect by using appropriate models.

In conclusion, in this large northwestern city with specific climatic and geographical characteristics, given its different demographic characteristics and types of air pollution, exploring the association between local air pollution and outpatient allergic conjunctivitis, especially, considering the possible lagged effects of allergic conjunctivitis owing to the ocular surface immune response, etc., would provide timely and comprehensive insights and policy decisions for local public health and eye disease prevention. Therefore, we conducted a time-series analysis of the lagging patterns of the six main air pollutants with allergic conjunctivitis in Urumqi, combining age, sex, seasonal subgroup analysis, and further detailed investigation of the types of exposure assessment (continuous and categorical exposure) for allergic conjunctivitis.

## 2 Materials and methods

### 2.1 Study area and outpatient visit data

Urumqi is the political, economic, and transportation center of Xinjiang, located in the northwest of China at the heart of Asia and Europe. As the world's most distant city from the sea (covering an area of approximately 12,000 square kilometers), it is called the “Capital of Asia” and is located about 2,500 kilometers from the ocean and coastline, with a unique regional population concentration and air pollution caused by industrialization in recent years.

Our outpatient visits were enrolled from electronic clinic data collected by the Ophthalmology Department of the First Affiliated Hospital of Xinjiang Medical University (Urumqi, Xinjiang, China) between January 1, 2013, and December 31, 2020. As the largest ophthalmology hospital in Xinjiang province, it is the most favorable and preferred destination for residents suffering from eye illnesses throughout Xinjiang, including the neighboring areas of Urumqi.

**Figure 1:**
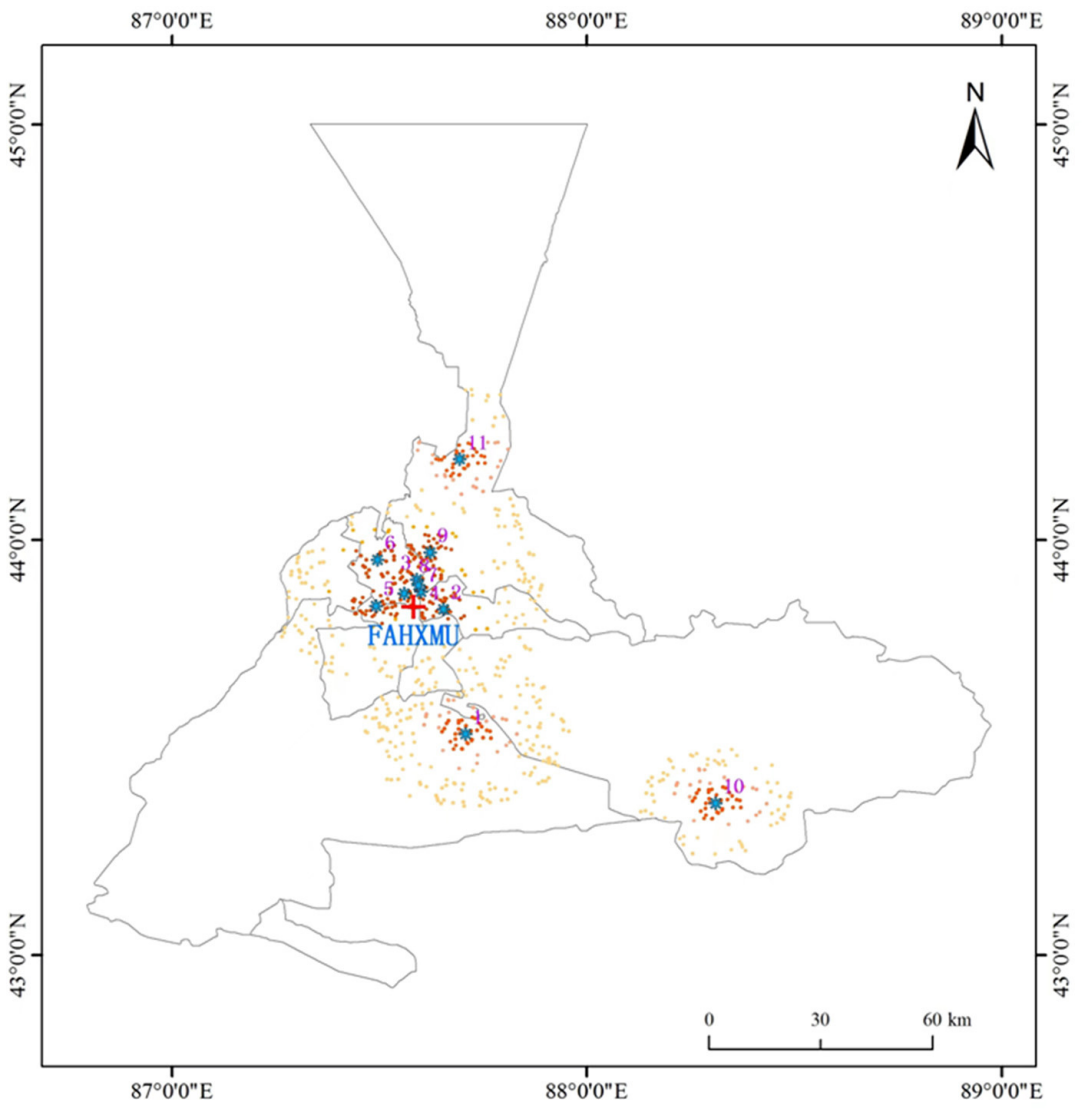
Locations of environmental monitoring stations and hospital in Urumqi, Xinjiang, China. The location of each air quality monitor is marked with a blue dot; the location of the hospital is marked with a red cross; the home address of each recruited patient is marked with a black dot. FAHXMU: First Affiliated Hospital of Xinjiang Medical University (Urumqi, Xinjiang, China); 1: Urumqi Toll House; 2: Urumqi Thirty-first High School; 3: Urumqi Xin Shi Da Hot Spring Campus; 4: Urumqi Hong-guang Mountain Area; 5: Urumqi Great Green Valley; 6: Urumqi Xinjiang Academy of Agricultural Sciences Farm; 7: Urumqi Monitoring Station; 8: Urumqi Railway Bureau; 9: Urumqi Mi-dong District Environmental Protection Bureau; 10: Urumqi Da-ban Cheng District Environmental Protection Bureau; Urumqi Training Base Protection Bureau; 11: Urumqi Training Base.

Basic biographical information, including date of visit, visit number, gender, age, and home residence (current place of living), was incorporated. In the present study, patients whose current place of residence were not in Urumqi and patients originating from urgent care were excluded, given their possible exposure-response error. Diagnosis of allergic conjunctivitis was performed according to the diagnostic codes of the tenth edition of the International Classification of Disease (ICD-10) (H10.101). More information on the hospital location coordinates for this study is available in Figure 1.

### 2.2 Air pollution data and covariate indicators

Six prominent air pollutants were included in this study. Specifically, 24-hourly aver-ages of PM_2.5_, PM_10_, CO, NO_2_, and SO_2_, and 8-hourly maximum values of O_3_ from 11 standard urban background fixed air quality monitors covering the central Urumqi region from January 1, 2013, to December 31, 2020, were collected. Meteorological data from the China Meteorological Data Sharing Service (http://data.cma.cn) for daily mean temperature (T, °C), daily relative humidity (RH, %), and atmospheric pressure (AP, hPa) were also included as covariates for adjustment. The latitude, longitude, and geographic distribution of the 11 air pollutant monitoring sites are summarized in Figure 1.

### 2.3 Statistical analysis

Utilize the R package “seasons” to set alternative values for the control groups in the model, and “days of the week (DOW)” was used as an indicator variable to reconcile long-term trends, seasonal pattern effects, and DOW effects. Spearman rank correlation coefficients were used to calculate the degree of correlation between air pollutants and meteorological factors. Previous studies have suggested a latency period of up to 5–6 days between air pollution exposure and the onset of conjunctivitis (12, 19), therefore, we developed a quasi-Poisson generalized linear regression model with a distributed lagged nonlinear model (DLNM) to investigate the lagged effects of air pollutants. Considering that daily outpatient visits for allergic conjunctivitis can be considered as rare events that approximate a quasi-Poisson distribution. Specifically, we performed separate calculations for single-day lags (lag 0 to lag 7) and cumulative daily lags (lag 0–1 to lag 0–7), defining lag 0 days as current-day exposure and a maximum lag of 7 days. The optimal lagged effect for each model is obtained from the maximum value of relative risk (RR) and the minimum value of P. Natural cubic spline curves (ns) with three degrees of freedom (df) were used to smoothly control for covariates, including meteorological factors such as T, RH, and AP. Simultaneously the df was determined according to the residual independence principle based on the minimum of the sum of the absolute values of the partial autocorrelation functions (PACF) underlying the model residuals.

Diagnostic data records for outpatient visits with missing baseline information were excluded first. We used Baidu Maps 0.A. to further filter out participants whose residence address was below the average distance of 20 km from the nearest air quality monitoring site and located in major areas of Urumqi city (including Tianshan District, Shaibak District, Xincheng District, Shuimogou District, Tutunhe District, Dabancheng District, Middong District, and Urumqi County) to ensure the representativeness of the results. The lagged effect model adjusted for meteorological factors is as follows:


$$\begin{array}{l} {\text{Y}}_{\text{t}}\sim \text{quasiPoisson }\left({\text{μ}}_{\text{t}}\right)\\ \text{Log}\left[\text{E}\left({\text{Y}}_{\text{t}}\right)\right]={\text{β}}^{*}{\text{Z}}_{\text{t}}+\text{factor }\left(\text{DOW}\right)\\ +\text{ns }\left(\text{time},\text{ df}=7/\text{year}\right)+\text{ns }\left(\text{N}{\text{O}}_{2},\text{ df}=3\right)\\ +\text{ns }\left({\text{O}}_{3},\text{ df}=3\right)+\text{ns }\left(\text{S}{\text{O}}_{2},\text{ df}=3\right)+\text{ns }\left(\text{CO},\text{ df}=3\right)\\ +\text{ns }\left(\text{P}{\text{M}}_{2.5},\text{ df}=3\right)+\text{ns }\left(\text{P}{\text{M}}_{10},\text{ df}=3\right)+\text{ns }\left(\text{T},\text{ df}=3\right)\\ +\text{ns }\left(\text{RH},\text{ df}=3\;\right)+\text{ns }\left(\text{AP},\text{ df}=3\right)+\text{intercept } \end{array}$$

Where E(Y_t_) represents the estimated number of outpatient visits for allergic conjunctivitis on day t; Z_t_ represents the concentration of a specific type of pollutant on day t; β and ns represent the exposure coefficient as well as the natural cubic spline function, respectively; df denotes the degrees of freedom; As mentioned, DOW refers to the “day of the week” indicator variable; T, RH, and AP represent the average air temperature, relative humidity, and atmospheric pressure separately. Further, we report effect values for RRs and 95% confidence intervals (CI) for continuous exposure (per interquartile range (IQR) increased compared to the mean exposure level) and categorical exposure (75%, 90%, 95%, and 99% compared to 25%), respectively, in this research.

For subgroup analyses, we used sex (male and female), age (0–18, 19–64, and ≥65 years), and season [warm season (April to September) and cold season (January to March and October to December)] to investigate potential influencing factors affecting the association between air pollution and the onset of allergic conjunctivitis. All statistical analyses were performed using R software version 4.2.2 (2022-10-31), and packages including “season” “dlnm” and “splines” were used. All P values in the statistical tests were based on two-sided tests and <0.05 was statistically significant. This experiment was con-ducted in agreement with the Declaration of Helsinki, and all processes and criteria were reviewed and authorized by the Medical Ethics Committee of the First Affiliated Hospital of Xinjiang Medical University.

## 3 Results

**Figure 2:**
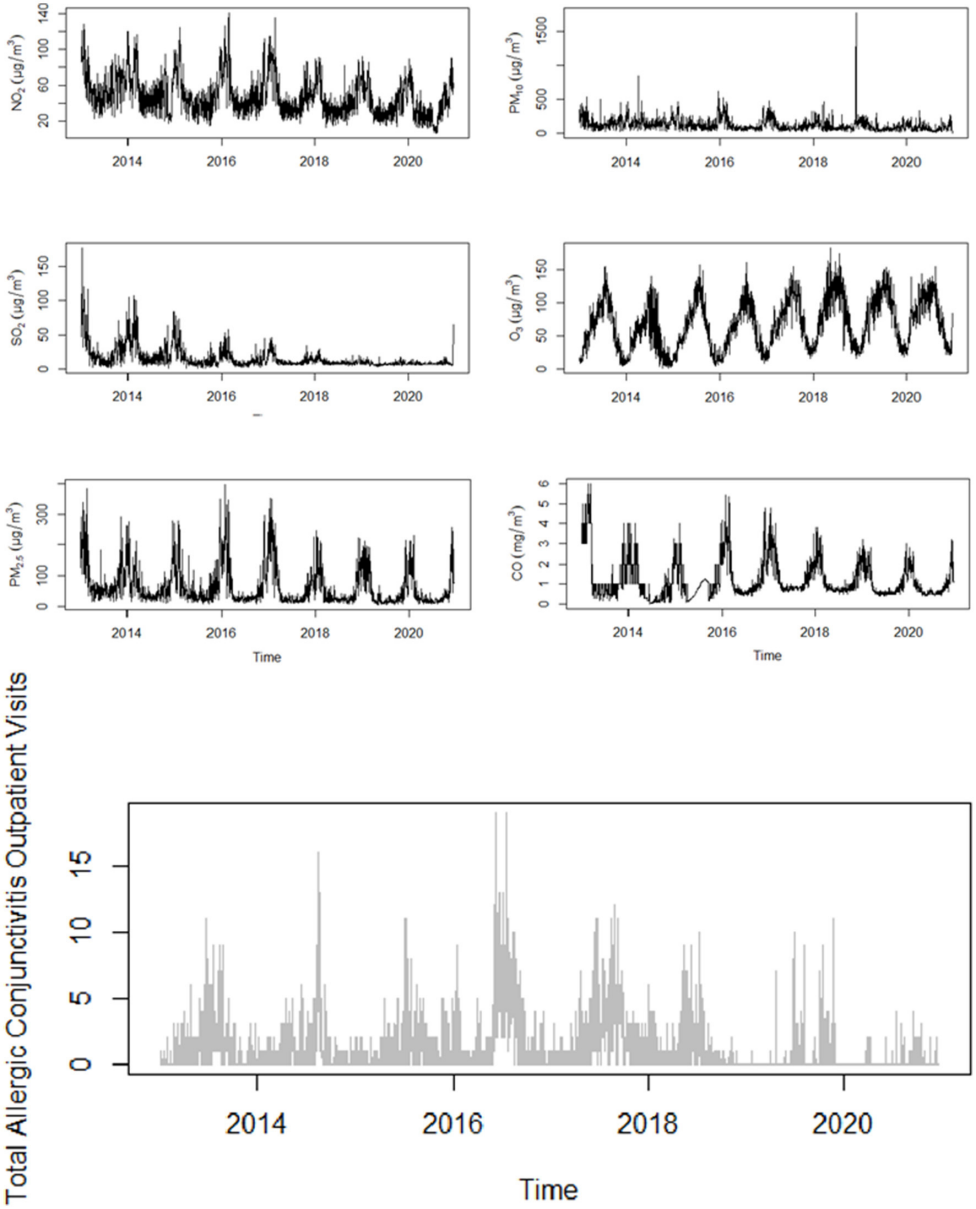
Distribution of air pollutants and outpatient visits for allergic conjunctivitis over time in Urumqi (2013–2020).

Table 1, Supplementary Figure S1 depicts the major six air pollutants, meteorological factors, and the basic characteristics of 3,325 eligible allergic conjunctivitis cases between 2013 and 2020. It can be seen in Figure 2 that the six air pollutants and the number of allergic conjunctivitis outpatient visits show a significant cyclical trend. CO, SO_2_, and O_3_ levels were below the Chinese national ambient air quality level 2 standard, while NO_2_, PM_2.5_, and especially PM_10_ levels significantly exceeded this standard. In addition, the results show significant climate change in the Urumqi region with a wide range of temperature variation (from −26.0 to 35.1°C), humidity variation (from 6.0 to 100.0%), and wind speed variation from 0.0 to 14.8 m/s. The daily averages of temperature, humidity, air pressure, and wind speed were 8.4°C, 55.5%, 912.7 hPa, and 2 m/s. The local meteorological, as well as air pollution characteristics, confirm the significance of our previous complaint for this region of study. The remaining descriptive analyses of indicators (standard deviation (SD), minimum, 25th and 75th percentile, median, and maximum) and the characteristics of the cycle variation are shown in Figure 2, Supplementary Figure S1. During the eight-year period 2013–2020, there were slightly more female patients (57.26%) than male patients diagnosed with allergic conjunctivitis, with patients aged 19–64 years (60.39%) occupying the majority of the age group. The number of allergic conjunctivitis outpatient visits was greater in the warm season (77.59%) than in the cool season.

**Table 1:** Characteristics of outpatient for allergic conjunctivitis in Urumqi (01/01/2013 to 12/31/2020).

**Variables**	**Number of measurements**	**Mean (SD)**	**Min**	**P25**	**Median**	**P75**	**Max**
**Air pollutant concentration**							
PM_2.5_ (μg/m^3^)	2,921	64.5 (61.9)	6	23	39	84	397
PM_10_ (μg/m^3^)	2,921	120.2 (90.8)	10	62	98	153	1,766
SO_2_ (μg/m^3^)	2,921	16 (15.2)	2	8	10	17	177
NO_2_ (μg/m^3^)	2,921	48.5 (21)	7	33	44	60	141
CO (mg/m^3^)	2,921	1.2 (1)	0.01	0.6	0.9	1.5	6
O_3_ (μg/m^3^)	2,921	68.2 (37.7)	2	35	67	99	182
**Meteorological factors**							
Mean temperature (°C)	2,921	8.4 (13.7)	−26	−4.5	10.7	20.6	35.1
Relative humidity (%)	2,921	55.5 (21.3)	6	37	54	74	100
Atmospheric pressure (hpa)	2,921	912.7 (7.8)	842	908	913	918	934
Wind speed (m/s)	2,921	2 (1.1)	0	1.4	1.9	2.4	14.8
**Number of conjunctivitis outpatient visits (n)**							
Total	3,325	1.1 (2.1)	0	0	0	1	19

SD, standard deviation; Min, minimum; P25, 25th percentile; P75, 75th percentile; Max, maximum.

**Figure 3:**
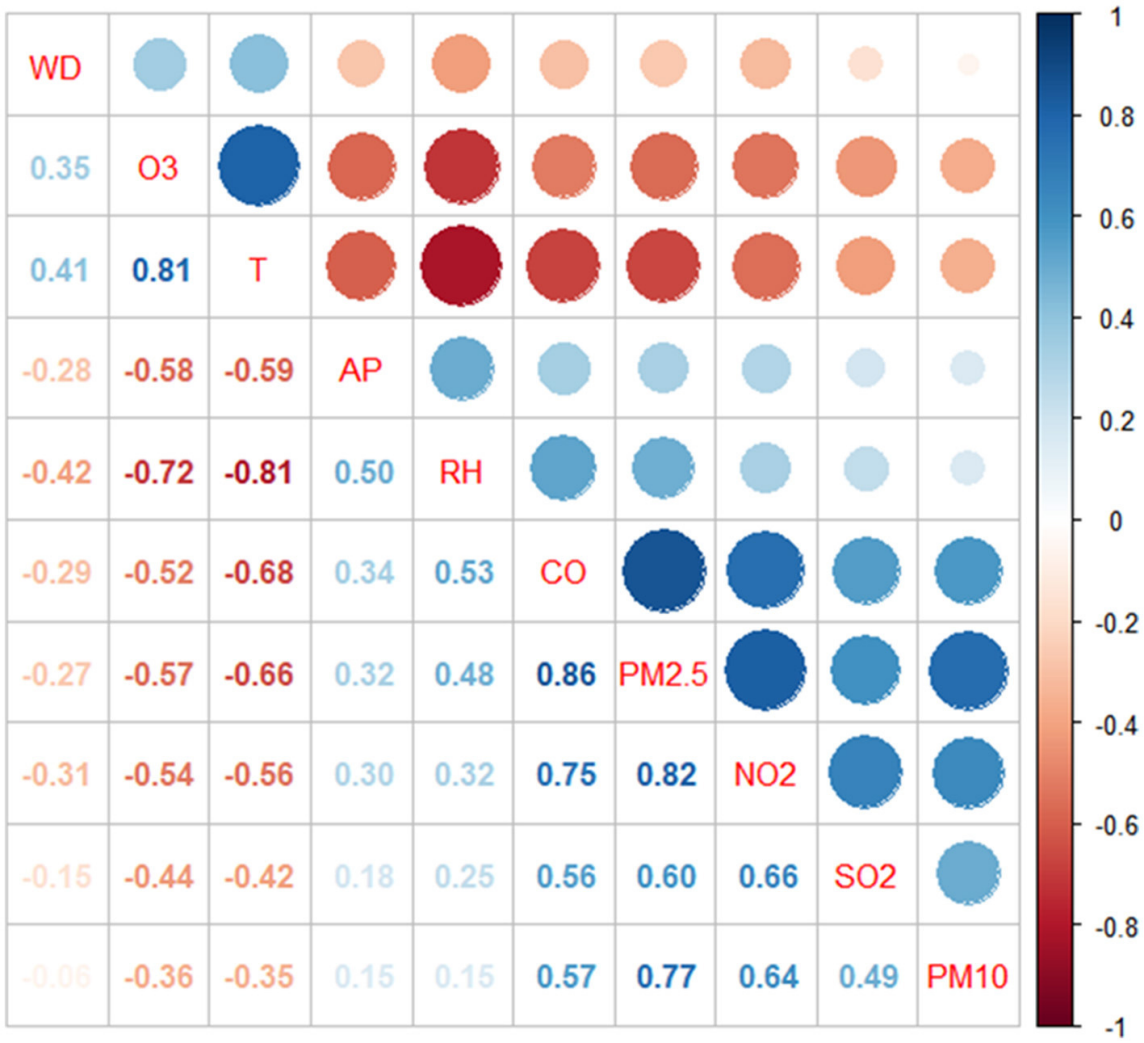
Spearman's correlation between different air pollutants and meteorological factors. T, Mean temperature; RH, Relative humidity; AP, Atmospheric pressure.

We report the correlation between each of the four main meteorological factors and the six air pollutants by Spearman analysis, as detailed in Figure 3. It can be seen therein that PM_2.5_ is highly correlated with CO (*r* = 0.86, *p* < 0.0001), NO_2_ (*r* = 0.82, *p* < 0.0001), and PM_10_ (r = 0.77, *p* < 0.0001) with correlation coefficients close to 0.8. The remaining pollutants were also correlated with each other or with meteorological factors to varying degrees (*p* < 0.0001).

**Figure 4:**
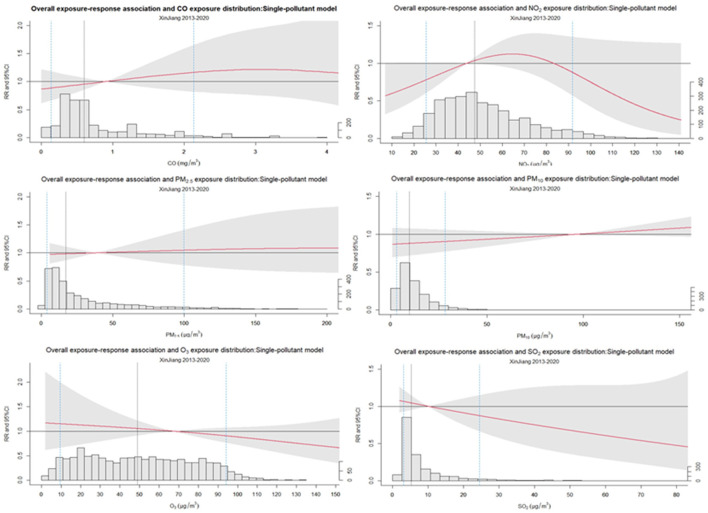
Overall exposure-response association for air pollutants and allergic conjunctivitis outpatient visits: single-pollutant model.

After the single-day lagged model analysis for each pollutant (Table 2, Figure 4), it can be found that when considering the continuous exposure analysis, for each IQR unit increase in concentration PM_10_ at lag2 (RR = 1.076, 95% CI:1.014–1.142) and NO_2_ at lag3 (RR = 1.061, 95% CI:1.002–1.124) were statistically significantly correlated with allergic conjunctivitis outpatient visits. When analyzing the air pollution concentrations for categorical exposure, on considering the effect of lag 2 days (Lag2), compared to 25th, PM_10_ in the 75th, 90th, 95th, and 99th were significantly associated with allergic conjunctivitis outpatient visits. Significant associations were found between 90th and 95th NO_2_ levels and allergic conjunctivitis risk only when considering the 3-day lag (lag3). No statistically significant associations were observed between the remaining pollutant concentrations and allergic conjunctivitis outpatient visits. The results of the multi-pollutant model analysis were similar to the single-pollutant results, and the cumulative day lag did not reveal any statistically significant association between pollutant levels and risk of allergic conjunctivitis, and no significant correlation was found for both continuous exposure (each additional IQR unit) and categorical exposure (75th, 90th, 95th and 99th percentile vs. 25th percentile) (Table 3, Supplementary Tables S1–S8, Supplementary Figures S2–S8).

**Table 2:** Lag effects of per IQR increase in air pollutants on allergic conjunctivitis: single-pollutant model.

**Lag effects**	**PM_2.5_**	**PM_10_**	**CO**	**SO_2_**	**NO_2_**	**O_3_**
**Single lag effects RRs (95% CI)**						
Lag 0	0.923 (0.748–1.138)	0.910 (0.804–1.030)	1.075 (0.878–1.315)	0.979 (0.867–1.104)	0.977 (0.833–1.147)	0.810 (0.631–1.040)
Lag 1	1.075 (0.945–1.222)	1.055 (0.986–1.129)	1.011 (0.882–1.158)	1.005 (0.935–1.080)	1.001 (0.907–1.105)	0.937 (0.802–1.094)
Lag 2	1.088 (0.971–1.219)	**1.076 (1.014–1.142)** ^*^	0.993 (0.884–1.116)	1.007 (0.945–1.073)	1.037 (0.952–1.129)	0.974 (0.856–1.110)
Lag 3	1.036 (0.958–1.121)	1.038 (0.994–1.083)	0.997 (0.918–1.084)	0.998 (0.954–1.044)	**1.061 (1.002–1.124)** ^*^	0.967 (0.878–1.066)
Lag 4	1.001 (0.911–1.099)	1.015 (0.965–1.068)	1.003 (0.907–1.110)	0.990 (0.939–1.044)	1.057 (0.985–1.134)	0.970 (0.870–1.083)
Lag 5	0.980 (0.910–1.056)	1.008 (0.968–1.050)	1.009 (0.934–1.091)	0.983 (0.943–1.025)	1.029 (0.974–1.088)	0.984 (0.900–1.076)
Lag 6	0.970 (0.904–1.041)	1.011 (0.971–1.051)	1.015 (0.945–1.091)	0.978 (0.940–1.018)	0.988 (0.938–1.041)	1.006 (0.920–1.099)
Lag 7	0.964 (0.838–1.110)	1.018 (0.944–1.098)	1.021 (0.88–1.185)	0.973 (0.900–1.052)	0.942 (0.847–1.048)	1.031 (0.877–1.212)
**Cumulative lag effects RRs (95% CI)**						
Lag 0–1	0.992 (0.804–1.223)	0.960 (0.842–1.095)	1.087 (0.888–1.329)	0.983 (0.866–1.117)	0.978 (0.835–1.146)	0.759 (0.565–1.020)
Lag 0–2	1.079 (0.861–1.353)	1.033 (0.900–1.186)	1.079 (0.862–1.350)	0.990 (0.862–1.137)	1.014 (0.853–1.205)	0.740 (0.529–1.036)
Lag 0–3	1.118 (0.886–1.411)	1.072 (0.929–1.236)	1.076 (0.857–1.351)	0.988 (0.855–1.141)	1.076 (0.900–1.287)	0.716 (0.501–1.022)
Lag 0–4	1.119 (0.872–1.435)	1.088 (0.934–1.268)	1.080 (0.849–1.373)	0.978 (0.838–1.140)	1.137 (0.939–1.377)	0.695 (0.476–1.013)
Lag 0–5	1.097 (0.835–1.440)	1.097 (0.928–1.296)	1.090 (0.839–1.416)	0.961 (0.815–1.135)	1.171 (0.949–1.444)	0.684 (0.458–1.022)
Lag 0–6	1.064 (0.806–1.405)	1.108 (0.932–1.317)	1.106 (0.854–1.434)	0.940 (0.795–1.112)	1.157 (0.934–1.434)	0.688 (0.455–1.038)
Lag 0–7	1.026 (0.763–1.380)	1.128 (0.938–1.357)	1.130 (0.865–1.477)	0.915 (0.769–1.089)	1.090 (0.867–1.370)	0.709 (0.459–1.096)

Bolded represents statistically significant correlation.

RRs, relative risks; ^*^P < 0.05.

**Table 3:** Lag effects of per IQR increase in air pollutants on allergic conjunctivitis: multi-pollutant model.

**Characteristics**	**PM**_**2.5**_		**PM**_**10**_		**CO**		**NO**_**2**_		**SO**_**2**_		**O**_**3**_	
	**Lag effects RRs (95% CI)**											
	**Single**	**Cumulative**	**Single**	**Cumulative**	**Single**	**Cumulative**	**Single**	**Cumulative**	**Single**	**Cumulative**	**Single**	**Cumulative**
Adjusted for PM_2.5_	–	–	**1.066 (1.003–1.133)** ^*^	1.213 (0.942–1.562)	1.158 (0.920–1.458)	1.187 (0.921–1.529)	1.059 (0.999–1.122)	1.231 (0.962–1.574)	0.977 (0.938–1.017)	1.283 (0.98–1.679)	0.814 (0.633–1.047)	0.671 (0.447–1.006)
Adjusted for PM_10_	1.079 (0.959–1.214)	1.190 (0.876–1.616)	–	–	1.158 (0.920–1.458)	1.155 (0.922–1.448)	1.076 (0.993–1.166)	1.212 (0.961–1.528)	0.977 (0.939–1.017)	0.922 (0.769–1.106)	0.814 (0.633–1.047)	0.671 (0.447–1.006)
Adjusted for CO	1.093 (0.976–1.225)	0.863 (0.672–1.106)	**1.078 (1.015–1.144)** ^*^	0.884 (0.769–1.015)	–	–	**1.064 (1.004–1.127)** ^*^	0.910 (0.750–1.105)	0.976 (0.938–1.016)	0.879 (0.729–1.059)	0.808 (0.629–1.037)	0.693 (0.475–1.013)
Adjusted for NO_2_	1.114 (0.962–1.29)	1.148 (0.881–1.494)	**1.080 (1.017–1.148)** ^*^	1.149 (0.940–1.404)	0.997 (0.887–1.121)	1.178 (0.926–1.500)	–	–	0.977 (0.938–1.017)	0.900 (0.740–1.094)	0.805 (0.626–1.034)	0.702 (0.481–1.025)
Adjusted for SO_2_	1.089 (0.970–1.221)	1.155 (0.905–1.473)	**1.081 (1.018–1.148)** ^*^	1.155 (0.954–1.398)	1.103 (0.894–1.360)	1.179 (0.893–1.557)	**1.061 (1.001–1.124)** ^*^	1.206 (0.961–1.514)	–	–	0.804 (0.625–1.034)	0.688 (0.470–1.007)
Adjusted for O_3_	1.096 (0.978–1.228)	0.888 (0.717–1.101)	**1.079 (1.017–1.146)** ^*^	0.889 (0.784–1.009)	1.016 (0.946–1.092)	1.107 (0.844–1.451)	**1.067 (1.007–1.13)** ^*^	1.182 (0.958–1.458)	0.983 (0.944–1.023)	0.933 (0.783–1.113)	–	–
Adjusted for the other five pollutants	1.090 (0.968–1.228)	1.164 (0.803–1.686)	**1.078 (1.013–1.148)** ^*^	1.255 (0.967–1.629)	1.092 (0.851–1.401)	1.178 (0.843–1.647)	**1.069 (1.008–1.133)** ^*^	1.182 (0.958–1.458)	0.978 (0.938–1.019)	0.933 (0.783–1.113)	0.798 (0.617–1.032)	0.663 (0.438–1.004)

Bolded represents statistically significant correlation.

RRs, relative risks; ^*^P < 0.05.

The results of the subgroup analysis are presented in Table 4. Subgrouping by gender, it can be seen that except for O_3_ and PM_10_, there were no gender differences between the remaining air pollutants and the risk of developing allergic conjunctivitis. Specifically, the increase in allergic conjunctivitis in females was significantly associated with a cumulative lag effect for per IQR unit increase in PM_10_ exposure (RR = 1.388, 95% CI:1.065–1.811), and the decrease in outpatient visits for allergic conjunctivitis in men was significantly associated with a cumulative lagged effect under O3 per increased IQR unit of exposure (RR = 0.540, 95% CI:0.317–0.923). In age-specific subgroup analyses, PM_2.5_ and O_3_ were significantly correlated with increased allergic conjunctivitis in the 0–1 years age group only. This was observed in both the single-day lag (for PM_2.5_, RR = 1.936; for PM_2.5_, RR = 3.718) as well as the cumulative lag (for PM_2.5_, RR = 5.571; for PM_2.5_, RR = 3.718). However, there was no significant correlation between any air pollutant and allergic conjunctivitis in the age group 2–5 yrs. PM_10_ showed a statistically significant correlation with allergic conjunctivitis visits in the age groups 0–1 years, 6–18 years, 19–64 years, and ≥65 years. Specifically, in the single-day lag (for 0–1 years, RR = 1.333; for 19–64 years, RR = 1.093; for ≥65 years, RR = 1.169) as well as in the cumulative lag (for 0–1 years, RR = 4.047; for 6–18 years, RR = 1.810; for ≥65 years, RR = 2.905) could both be observed. NO_2_ was only significantly associated with single-day lags for 19-64 years (RR = 1.072) and ≥65 years (RR = 1.205). SO_2_ was only significantly associated with single-day lag for ≥65 years (RR = 1.217) of allergic conjunctivitis outpatient visits. The warm and cold subgroup results showed that allergic conjunctivitis outpatient visits were more sensitive to exposure to air pollutants in the warm season. Like the Table 3 results, there was a statistically significant association between PM_10_ (RR = 1.100, 95% CI:1.025–1.181) as well as NO_2_ (RR = 1.730, 95% CI:1.074–2.787) and allergic conjunctivitis.

**Table 4:** Lag effects of per IQR increase in air pollutant on allergic conjunctivitis with subgroup analysis.

**Characteristics**	**PM**_**2.5**_		**PM**_**10**_		**CO**		**NO**_**2**_		**SO**_**2**_		**O**_**3**_	
	**Lag effects RRs (95% CI)**											
	**Single**	**Cumulative**	**Single**	**Cumulative**	**Single**	**Cumulative**	**Single**	**Cumulative**	**Single**	**Cumulative**	**Single**	**Cumulative**
**Sex**												
Male	1.113 (0.932–1.331)	1.174 (0.718–1.920)	1.057 (0.970–1.153)	0.917 (0.694–1.212)	0.943 (0.844–1.055)	1.220 (0.843–1.765)	1.039 (0.958–1.126)	1.168 (0.825–1.654)	0.968 (0.913–1.027)	1.057 (0.843–1.327)	0.782 (0.565–1.083)	**0.540 (0.317–0.923)** ^*^
Female	1.080 (0.985–1.184)	1.227 (0.789–1.908)	1.057 (0.970–1.153)	**1.388 (1.065–1.811)** ^*^	1.040 (0.944–1.145)	1.275 (0.866–1.877)	1.039 (0.958–1.126)	1.354 (0.999–1.834)	0.976 (0.931–1.023)	0.941 (0.748–1.183)	0.782 (0.565–1.083)	0.812 (0.598–1.102)
**Age (years)**												
0–1	**1.936 (1.094–3.428)** ^*^	**5.571 (1.951–15.904)** ^**^	**1.333 (1.105–1.608)** ^**^	**4.047 (1.521–10.769)** ^**^	0.055 (0.007–0.411)	0.055 (0.007–0.411)	0.147 (0.033–0.657)	0.078 (0.012–0.481)	0.976 (0.931–1.023)	0.767 (0.312–1.890)	**3.718 (1.433–9.643)** ^**^	**3.718 (1.433–9.643)** ^**^
2–5	1.181 (0.913–1.527)	1.667 (0.704–3.947)	1.086 (0.922–1.28)	0.767 (0.441–1.336)	0.847 (0.578–1.242)	1.925 (0.899–4.126)	0.578 (0.321–1.038)	0.578 (0.321–1.038)	1.094 (0.885–1.351)	1.132 (0.747–1.716)	0.591 (0.342–1.021)	0.262 (0.113–0.607)
6–18	1.091 (0.895–1.331)	0.793 (0.439–1.433)	1.126 (0.990–1.280)	**1.810 (1.164–2.815)** ^**^	1.186 (0.778–1.806)	1.925 (0.899–4.126)	1.198 (0.843–1.703)	1.247 (0.815–1.908)	0.947 (0.877–1.022)	0.745 (0.540–1.029)	0.910 (0.774–1.069)	0.547 (0.282–1.061)
19–64	0.956 (0.879–1.040)	1.247 (0.805–1.934)	**1.093 (1.012–1.179)** ^*^	1.182 (0.908–1.537)	1.086 (0.911–1.295)	1.238 (0.833–1.840)	**1.072 (1.001–1.148)** ^*^	1.312 (0.960–1.794)	0.974 (0.926–1.024)	0.945 (0.737–1.212)	0.858 (0.627–1.173)	0.858 (0.627–1.173)
≥65	0.666 (0.229–1.935)	0.595 (0.185–1.909)	**1.169 (1.023–1.336)** ^*^	**2.905 (1.233–6.844)** ^*^	**1.242 (1.006–1.534)** ^*^	0.653 (0.262–1.626)	**1.205 (1.031–1.410)** ^*^	2.104 (0.913–4.847)	**1.217 (1.053–1.406)** ^**^	1.777 (0.987–3.201)	0.421 (0.170–1.042)	0.404 (0.086–1.905)
**Season**												
Warm (April to September)	1.046 (0.967–1.132)	0.908 (0.715–1.154)	**1.100 (1.025–1.181)** ^**^	0.828 (0.603–1.137)	1.036 (0.988–1.087)	0.925 (0.776–1.102)	1.068 (0.980–1.165)	**1.730 (1.074–2.787)** ^*^	1.021 (0.967–1.078)	1.043 (0.882–1.233)	0.669 (0.492–0.910)	0.497 (0.314–0.785)
Cold (October to March)	0.881 (0.807–0.962)	1.386 (0.769–2.498)	0.962 (0.906–1.021)	0.808 (0.530–1.234)	0.878 (0.803–0.961)	0.548 (0.326–0.921)	0.751 (0.625–0.902)	0.620 (0.389–0.986)	0.814 (0.687–0.965)	0.704 (0.379–1.305)	0.781 (0.626–0.975)	0.497 (0.314–0.785)

Bolded represents statistically significant correlation.

RRs, relative risks; ^*^P < 0.05, ^**^P < 0.01.

Sensitivity analysis was performed for the two-pollutant and multi-pollutant models, and the results in Supplementary Table S7 were like the previous results, suggesting the stability of our study results.

## 4 Discussion

To our knowledge, this is the first study conducted so far that focuses on the potential association and lagged effect of air pollutants (PM_2.5_, PM_10_, CO, NO_2_, SO_2_, O_3_) with outpatient visits for allergic conjunctivitis in the large city of Urumqi, Xinjiang, China, confirming the underlying lagged effect of air pollution on allergic conjunctivitis. Our results showed that there were significant positive associations between elevated PM_10_ and NO_2_ levels and increased allergic conjunctivitis outpatient visits with a 2-day lag and a 3-day lag, respectively, and the results of the subgroup analysis further indicated that it was more prominent in the warm season. Females were more sensitive to PM_10_ exposure. Infant (0–1 year) allergic conjunctivitis was significantly associated with particulate pollutants (PM_2.5_, PM_10_) as well as O_3_, and older people over 65 years of age appeared to be more susceptible to the effects of air pollution.

As previously described, there is limited evidence and inconsistent findings regarding the association between air pollution and allergic conjunctivitis (10, 11, 16, 17, 20–23). A regional study conducted by Cheng-Wei Lu et al. in Northeast China covering 20 prefectures between 2014 and 2018 confirmed that atmospheric pollutants including PM_2.5_, PM_10_, SO_2_, NO_2_, CO, and O3 were positively associated with outpatient visits of allergic conjunctivitis (11). An earlier published 5-year short-term study by Jia-xu Hong et al. based on a time-series analysis in Shanghai found that NO_2_ and O_3_ were significantly associated with outpatient visits for allergic conjunctivitis, but the associations of PM_10_, PM_2.5_, SO_2_ and allergic conjunctivitis were statistically marginal (17). In fact, the prevalence of allergic conjunctivitis has significant regional variations, even in different parts of the same country (e.g., northeast, and southeast China). On the other hand, Shanghai, as a representative coastal port city in southeast China, has a mild and humid climate, whereas the northeast region is relatively dry and cold, and the different climatic conditions and effects of atmospheric particulate matter in the two regions may explain the inconsistent findings.

Our findings reveal the significant association between increased NO_2_ levels and elevated risk of outpatient visits for allergic conjunctivitis, which supports the findings of several previous studies, such as Fu et al. (24), Lu et al. (25) and Wang et al. (26) NO_2_ is recognized as an important atmospheric allergen that can increase the risk of developing allergic diseases (e.g., respiratory, etc.). Ulrike Gehring et al. reported the results of data from a Dutch Prevention and Incidence of Asthma and Mite Allergy (PIAMA) birth cohort containing 3,687 participants which suggested that early childhood exposure to motor vehicle exhaust pollution containing high levels of NOx may have long-term effects on the development of asthma (27). Regarding the mechanism of NO_2_ as an allergen, the conjunctiva, as the site of direct contact with air, NO_2_ can induce chemical modifications that directly disrupt the tissue surface or mucosal barrier and can even indirectly lead to an imbalance of the conjunctival internal environment by enhancing the allergic response to other allergens. In addition, as a major component of traffic exhaust mixtures, particularly affects urban residents, and its ability to oxidize and acidify tear fluid in the ocular surface tissues is an important cause of allergic conjunctivitis among populations in developing industrial areas (28).

As a respirable particulate matter, PM_10_ has similarly been reported to contain complex allergens such as dust, pollen, and mold, which may induce the development of ocular allergy symptoms through antigen-specific reactions. Jin Zhou et al. conducted a time-stratified case-crossover study at the Women and Children's Medical Center in Guangzhou City between 2016 and 2018, which suggested that per 10 μg/m^3^ increase in daily PM_10_ concentration was associated with a 1.3% (95% CI: 1.007–1.020) increase in the estimated risk of daily allergic conjunctivitis outpatient visits in children (20). In addition, Dai Miyazaki et al. published a web-based questionnaire using air pollutant data from the National Institute for Environmental Studies between 2012 and 2016 and multivariate logistic regression analysis, which suggested that the prevalence of AKC in Japan was significantly associated with levels of nitrogen dioxide, while the prevalence of VKC was significantly associated with levels of nitrogen oxides and PM_10_ (ORs of 1.72 and 1.54, respectively) (21). In addition, Hong et al. (17) and Lu et al. (11) also reported that higher ambient PM_10_ levels increased the likelihood of outpatient visits for allergic conjunctivitis. However, subgroup analyses suggest that relevant findings remain inconsistent in degree and direction, e.g., Zhou et al. reported that children aged 1–6 years in Guangzhou, China, were more sensitive to particulate pollutants (PM) exposure (20), whereas our results suggest that middle-aged and young adults aged 19–64 years in Urumqi suffering from allergic conjunctivitis are more susceptible to PM_10_ and NO_2_. This may be due to the more frequent outdoor exposure of the middle-aged population in the region relative to younger and older age groups resulting in more exposure to PM_10_. Increased public health education and preventive protection measures for ocular surface diseases could therefore be considered for people aged 19–64, who are also the main labor force and social value providers, to reduce the burden of disease and patient suffering.

Subgroup analyses found females were more sensitive to PM_10_ exposure. Whereas this finding is like the general findings of studies on outdoor air pollution and conjunctivitis, a case-crossover study in Hangzhou observed a significant association between outpatient conjunctivitis and PM_10_ and NO_2_ only in women (24), and short-term studies in Hefei and Guangzhou supported this finding (12, 20). In contrast, a time-series study by Zhou et al. in Guangzhou between 2016 and 2018 suggested that the risk of allergic conjunctivitis in girls and children aged 1–6 years was more sensitive to PM exposure and was more apparent in autumn and winter (20). A 5-year time-series study in Tai' an also found that male patients were more sensitive to air pollution (13). This contradiction may be since the local average daily PM_10_ concentrations in Guangzhou and Tai' an (56.3 μg/m^3^ and 109.9 μg/m^3^ respectively) were significantly lower than those in Xinjiang (120 μg/m^3^), which may have biased the results. We also found significant associations between allergic conjunctivitis in older people over 65 years old for a variety of air pollutants, including PM_10_ and NO_2_. As for the occurrence or exacerbation of adverse outcomes in older people exposed to air pollution, some previous studies have provided convincing evidence. Post-retirement outdoor activity in older Chinese people occurs more often in the morning and evening. These periods always coincide with periods of relatively high air pollutant concentrations. In addition, the reduced immunity and physical fitness of the older people allow further deposition of particulate matter, inducing the onset of ocular surface inflammation (29–31). In addition, we note that exposure to PM_10_ and NO_2_ during the warmer months promotes a high risk of outpatient allergic conjunctivitis, which can be explained by the increased exposure of the eyes to airborne pollutants during outdoor activities in warm weather. Interestingly, our study showed a negative association between elevated PM_2.5_, O3, and NO_2_ levels and the number of allergic conjunctivitis visits in cold weather, and it was the only negative association that occurred. This is inconsistent with the findings of some published studies, such as Zhou et al. (20) and Cheng et al. (14) whose results suggest that air pollutants have a stronger effect in cold weather. This may be due to the greater temperature variation throughout the year in Xinjiang compared to coastal or inland areas, with the difference between the coldest and warmest daily mean temperatures reaching up to 61.1 degrees, which may lead to reduced outdoor activity of the population in extreme climates, thus interfering with the interpretation of the results. From the mechanistic point of view, the variability in subgroups of the association of different air pollutants with allergic conjunctivitis may be related to the different pathogenesis and epidemiological patterns of individuals, leading to the variation in results.

Multiple mechanisms can account for the potential contribution of environmental pollution and allergic conjunctivitis. First, airborne PM can be classified in terms of their diameter: PM_10_ has a diameter of 2–10 μm, and PM_2.5_ has a diameter of 0.5–2 μm. PM_10_ consists mainly of dust, pollen, and mold, while PM_2.5_ includes combustion particulate matter, organic compounds, and metals. PM_10_ consists of many possible allergens or adjuvants, suggesting that respirable particulate points can supply air pollutants by providing antigenic specificity in response (32). It has also been observed that PM_2.5_ triggers allergic conjunctivitis through serum induced IgE production, mast cell, and eosinophil infiltration of the conjunctiva (33). Direct exposure of the human eye to particulate pollutants suspended in the air, which are blended with metal compounds such as nickel, aluminum, silicon, and titanium dioxide, has been shown to cause disruption of the lacrimal gland membrane and subsequently predispose to conjunctival pathology, including conjunctivitis (34). In addition, air pollution particulate matter may directly induce a range of adverse reactions, such as corneal epithelial damage, tissue inflammation, and oxidative stress by promoting external bacterial infections (35) and epigenetic changes, including DNA methylation (36). In the secondary, it has been suggested that exposure to diesel exhaust particles increases the expression of human conjunctival cytokines and adhesion molecules and induces increased expression of LARC in human conjunctival epithelial cells through stimulation of TNF and NF-kB, which recruit T cells and dendritic cells via chemokine receptors (CCR) 6, CCR36, and CCR37, thereby inducing conjunctival inflammation (37). On the other hand, oxidant contamination is normally caused by NO produced by automobiles, which is converted to NO_2_, followed by the production of O_3_. These contaminants damage the ocular surface either by lowering the pH of the tear fluid or directly through oxidation. These promote allergen penetration, induce inflammation, promote cytokine release, and lead to mucosal surface damage. Hot weather, strong winds, and the spread of pollen allergens can reinforce these effects, leading to instability of the ocular surface (38). It has also been shown that maternal NO_2_ exposure during pregnancy is associated with methylation of CpG sites in DNA neonatal mitochondria-related genes, which would affect the intracellular inflammatory response (39). Interestingly, by increasing endothelial cell oxidative stress, NO_2_ can impair endothelial cell vasodilation. NO_2_ acts as a strong oxidant and can lead to elevated levels of inflammatory factors in tears, such as IL-6, IL-1β, IFN-γ, and IL-17 (40). NO_2_, an allergy-related pollutant, can indirectly induce conjunctival inflammation by chemically altering and interfering with allergic responses (41). The exact mechanism is also present in O_3_ exposure, such as intense oxidative stress of O_3_ leading to conjunctival inflammation (42).

Overall, this study has several strengths. Firstly, we are the first long-term time-series analysis design conducted in Urumqi, the largest city in northwest China and the furthest from the ocean. Second, our study provides new population-based evidence, confirming for the first time the effect of PM_10_ and NO_2_ on the risk of outpatient visits for allergic conjunctivitis in Urumqi, and revealing their potential lagged effects. Thirdly, we conducted subgroup analyses to examine in detail the differences in the effects of gender and age as well as cold and warm seasonal factors among local allergic conjunctivitis patients in Urumqi.

Several limitations of this study are as follows, first, although the ophthalmology department of the First Affiliated Hospital of Xinjiang Medical University is the largest ophthalmology clinic in Xinjiang, considering the existence of a few other ophthalmology clinics in Urumqi, the omitted patients may lead to an underestimation of the effects of air pollution. Second, we mainly focused on outdoor air pollution levels, so indoor air pollution, including cooking fumes, was not available for analysis, and in addition, individual-level exposures were not available, although we used a series of methods to screen for patients living in major areas of Urumqi. For example, the joint use of current addresses and zip codes, and monitoring stations are also closest to individual addresses, and the future implementation of individual-based mobile monitors may address this issue. Third, other potential confounders such as pollen, UV light, volatile chemicals, and other allergens may also be present, and further retrospective analysis, as well as questionnaire surveys, may be necessary. In addition, although our study is the first to address the association between outdoor air pollution and allergic conjunctivitis in the Urumqi region, future studies should be conducted in further large-scale multicenter trials as well as long-period follow-up surveys in a wider variety of populations.

## 5 Conclusion

We conducted the first time-series study in Urumqi, Northwest China, which is also the world's farthest inland city from the ocean, including 3,325 allergic conjunctivitis outpatient visits from January 1, 2013, to December 31, 2020. Single-pollutant and multi-pollutant models were constructed to analyze the association between six major air pollutants and allergic conjunctivitis outpatient visits in Urumqi, and single-day and cumulative lag effects were further investigated in detail. Our study demonstrated for the first time that elevated PM_10_ and NO_2_ levels were significantly associated with an increase in allergic conjunctivitis outpatient visits with a 2-day and 3-day lag, respectively, and the results of the subgroup analysis further suggest that the effects of PM_10_ and NO_2_ on allergic conjunctivitis are more prominent in the warm season. Women are more sensitive to PM_10_ exposure and the effect of air pollution on allergic conjunctivitis is affected by age (such as infancy and the older people). Considering that the effects of particulate pollutants and NO_2_ have received increasing attention in this rapidly industrializing region, our study will provide further insight into the need to implement specific outdoor air pollution controls such as the burning of fossil fuels like coal, as well as contribute to special population protection policies.

## Publishers' note

All claims expressed in this article are solely those of the authors and do not necessarily represent those of their affiliated organizations, or those of the publisher, the editors and the reviewers. Any product that may be evaluated in this article, or claim that may be made by its manufacturer, is not guaranteed or endorsed by the publisher.

## Data availability statement

The original contributions presented in the study are included in the article/Supplementary material, further inquiries can be directed to the corresponding author.

## Ethics statement

The studies involving humans were approved by the Medical Ethics Committee of the First Affiliated Hospital of Xinjiang Medical University. The studies were conducted in accordance with the local legislation and institutional requirements. The participants provided their written informed consent to participate in this study. Written informed consent was obtained from the individual(s) for the publication of any potentially identifiable images or data included in this article.

## Author contributions

DL: Conceptualization, Formal analysis, Funding acquisition, Project administration, Resources, Writing – original draft. SG: Data curation, Formal analysis, Methodology, Writing – original draft. XW: Methodology, Writing – original draft. QW: Data curation, Methodology, Writing – original draft. JQ: Methodology, Writing – original draft. FT: Investigation, Validation, Writing – original draft. LT: Investigation, Validation, Writing – original draft. ZJ: Data curation, Visualization, Writing – original draft. XY: Investigation, Writing – review & editing.
