# Supplementary material for: Long-term effects of air pollution on daily outpatient visits for allergic conjunctivitis from 2013 to 2020: a time-series study in Urumqi, China
Source: Front Public Health. 2024 Oct 25;12:1325956. doi: 10.3389/fpubh.2024.1325956 (PMC11543485; doi:10.3389/fpubh.2024.1325956)

Supplementary Material

Long-term effects of air pollution on daily outpatient visits for allergic conjunctivitis from 2013 to 2020: a time-series study in Urumqi, China

Dongwei Liu, Siyu Gui, Xinchen Wang, Qianqian Wang, Jianchao Qiao, Fangbiao Tao, Zhengxuan Jiang, Xianglong Yi^*^

*** Correspondence:** Xianglong Yi: Gwhisper666@gmail.com

**Table S1.** Relative risks (RRs) of allergic conjunctivitis outpatient visits for PM_2.5_ (75th, 90th, 95th and 99th percentiles) exposure at various lag days, with the reference value of 25th percentile.

| Lag effects | 75th percentile | 90th percentile | 95th percentile | 99th percentile |
| --- | --- | --- | --- | --- |
| **Single lag effects RR (95% CI)** | | | | |
| Lag 0 | 0.924(0.741-1.152) | 0.838(0.559-1.256) | 0.774(0.475-1.264) | 0.674(0.344-1.320) |
| Lag 1 | 1.078(0.942-1.234) | 1.148(0.897-1.470) | 1.171(0.869-1.579) | 1.148(0.759-1.736) |
| Lag 2 | 1.091(0.968-1.229) | 1.185(0.952-1.476) | 1.236(0.948-1.613) | 1.275(0.882-1.843) |
| Lag 3 | 1.036(0.954-1.126) | 1.078(0.928-1.253) | 1.107(0.926-1.325) | 1.152(0.898-1.478) |
| Lag 4 | 0.999(0.906-1.103) | 1.006(0.840-1.204) | 1.019(0.820-1.266) | 1.056(0.782-1.425) |
| Lag 5 | 0.979(0.905-1.058) | 0.965(0.838-1.111) | 0.966(0.817-1.143) | 0.990(0.787-1.246) |
| Lag 6 | 0.969(0.899-1.044) | 0.943(0.826-1.077) | 0.935(0.802-1.090) | 0.942(0.767-1.157) |
| Lag 7 | 0.963(0.831-1.117) | 0.930(0.710-1.218) | 0.914(0.662-1.262) | 0.904(0.583-1.402) |
| **Cumulative lag effects RR (95% CI)** | | | | |
| Lag 0-1 | 0.996(0.798-1.243) | 0.962(0.647-1.431) | 0.907(0.568-1.447) | 0.773(0.406-1.472) |
| Lag 0-2 | 1.087(0.855-1.381) | 1.140(0.745-1.744) | 1.121(0.684-1.836) | 0.986(0.501-1.941) |
| Lag 0-3 | 1.126(0.879-1.442) | 1.229(0.796-1.898) | 1.241(0.755-2.040) | 1.135(0.578-2.232) |
| Lag 0-4 | 1.125(0.863-1.467) | 1.237(0.778-1.966) | 1.265(0.747-2.141) | 1.199(0.589-2.439) |
| Lag 0-5 | 1.102(0.824-1.472) | 1.194(0.719-1.981) | 1.222(0.688-2.172) | 1.187(0.548-2.571) |
| Lag 0-6 | 1.067(0.793-1.437) | 1.126(0.674-1.880) | 1.143(0.646-2.024) | 1.119(0.522-2.399) |
| Lag 0-7 | 1.028(0.748-1.413) | 1.047(0.609-1.799) | 1.045(0.579-1.885) | 1.011(0.463-2.208) |

**Table S2.** Relative risks (RRs) of allergic conjunctivitis outpatient visits for PM_10_ (75th, 90th, 95th and 99th percentiles) exposure at various lag days, with the reference value of 25th percentile.

| Lag effects | 75th percentile | 90th percentile | 95th percentile | 99th percentile |
| --- | --- | --- | --- | --- |
| **Single lag effects RR (95% CI)** | | | | |
| Lag 0 | 0.910(0.799-1.036) | 0.842(0.674-1.053) | 0.795(0.599-1.056) | 0.687(0.442-1.070) |
| Lag 1 | 1.055(0.984-1.131) | 1.103(0.975-1.247) | 1.139(0.971-1.335) | 1.233(0.966-1.575) |
| Lag 2 | 1.076(1.012-1.143)^*^ | 1.143(1.026-1.273)^*^ | 1.194(1.038-1.374)^*^ | 1.336(1.076-1.658)^*^ |
| Lag 3 | 1.037(0.992-1.085) | 1.070(0.989-1.157) | 1.094(0.989-1.210) | 1.161(0.995-1.355) |
| Lag 4 | 1.015(0.964-1.069) | 1.028(0.938-1.127) | 1.038(0.922-1.169) | 1.065(0.888-1.278) |
| Lag 5 | 1.008(0.966-1.051) | 1.015(0.943-1.092) | 1.020(0.927-1.121) | 1.033(0.893-1.194) |
| Lag 6 | 1.011(0.970-1.053) | 1.019(0.949-1.095) | 1.025(0.935-1.124) | 1.039(0.902-1.197) |
| Lag 7 | 1.018(0.942-1.101) | 1.033(0.900-1.185) | 1.043(0.873-1.246) | 1.065(0.808-1.403) |
| **Cumulative lag effects RR (95% CI)** | | | | |
| Lag 0-1 | 0.960(0.836-1.103) | 0.928(0.733-1.176) | 0.905(0.673-1.219) | 0.848(0.536-1.341) |
| Lag 0-2 | 1.033(0.893-1.196) | 1.061(0.827-1.361) | 1.081(0.790-1.480) | 1.132(0.702-1.827) |
| Lag 0-3 | 1.072(0.921-1.247) | 1.135(0.878-1.467) | 1.183(0.857-1.634) | 1.314(0.808-2.139) |
| Lag 0-4 | 1.088(0.925-1.280) | 1.167(0.886-1.537) | 1.228(0.869-1.735) | 1.400(0.834-2.351) |
| Lag 0-5 | 1.097(0.919-1.309) | 1.184(0.876-1.599) | 1.252(0.859-1.826) | 1.446(0.822-2.541) |
| Lag 0-6 | 1.108(0.922-1.332) | 1.206(0.884-1.647) | 1.284(0.869-1.896) | 1.502(0.842-2.680) |
| Lag 0-7 | 1.129(0.928-1.372) | 1.246(0.894-1.737) | 1.339(0.883-2.030) | 1.599(0.862-2.967) |

*: P＜0.05

**Table S3.** Relative risks (RRs) of allergic conjunctivitis outpatient visits for CO (75th, 90th, 95th and 99th percentiles) exposure at various lag days, with the reference value of 25th percentile.

| Lag effects | 75th percentile | 90th percentile | 95th percentile | 99th percentile |
| --- | --- | --- | --- | --- |
| **Single lag effects RR (95% CI)** | | | | |
| Lag 0 | 1.088(0.880-1.344) | 1.131(0.740-1.728) | 1.104(0.654-1.862) | 0.822(0.329-2.055) |
| Lag 1 | 1.008(0.871-1.165) | 1.036(0.784-1.369) | 1.066(0.763-1.490) | 1.231(0.667-2.271) |
| Lag 2 | 0.986(0.871-1.115) | 1.008(0.792-1.283) | 1.050(0.783-1.408) | 1.353(0.802-2.283) |
| Lag 3 | 0.992(0.908-1.085) | 1.010(0.853-1.196) | 1.041(0.851-1.273) | 1.245(0.851-1.822) |
| Lag 4 | 1.001(0.899-1.116) | 1.013(0.825-1.244) | 1.027(0.803-1.314) | 1.104(0.692-1.763) |
| Lag 5 | 1.011(0.930-1.100) | 1.015(0.866-1.189) | 1.009(0.836-1.219) | 0.960(0.677-1.360) |
| Lag 6 | 1.021(0.945-1.104) | 1.015(0.880-1.171) | 0.989(0.837-1.169) | 0.823(0.597-1.134) |
| Lag 7 | 1.032(0.879-1.211) | 1.016(0.752-1.371) | 0.968(0.677-1.385) | 0.701(0.345-1.423) |
| **Cumulative lag effects RR (95% CI)** | | | | |
| Lag 0-1 | 1.096(0.885-1.357) | 1.172(0.775-1.772) | 1.177(0.715-1.935) | 1.012(0.426-2.406) |
| Lag 0-2 | 1.080(0.849-1.374) | 1.180(0.750-1.859) | 1.235(0.724-2.107) | 1.369(0.544-3.450) |
| Lag 0-3 | 1.071(0.839-1.369) | 1.193(0.754-1.886) | 1.285(0.754-2.192) | 1.705(0.706-4.116) |
| Lag 0-4 | 1.073(0.828-1.390) | 1.209(0.745-1.961) | 1.320(0.753-2.315) | 1.882(0.753-4.705) |
| Lag 0-5 | 1.085(0.819-1.438) | 1.227(0.725-2.076) | 1.333(0.723-2.457) | 1.807(0.660-4.944) |
| Lag 0-6 | 1.108(0.838-1.466) | 1.245(0.742-2.091) | 1.319(0.727-2.391) | 1.487(0.571-3.875) |
| Lag 0-7 | 1.144(0.854-1.530) | 1.265(0.746-2.144) | 1.277(0.704-2.317) | 1.042(0.388-2.796) |

**Table S4.** Relative risks (RRs) of allergic conjunctivitis outpatient visits for NO_2_ (75th, 90th, 95th and 99th percentiles) exposure at various lag days, with the reference value of 25th percentile.

| Lag effects | 75th percentile | 90th percentile | 95th percentile | 99th percentile |
| --- | --- | --- | --- | --- |
| **Single lag effects RR (95% CI)** | | | | |
| Lag 0 | 1.012(0.861-1.189) | 0.968(0.744-1.258) | 0.913(0.623-1.338) | 0.809(0.417-1.571) |
| Lag 1 | 0.998(0.904-1.101) | 1.001(0.851-1.177) | 1.006(0.791-1.279) | 1.017(0.666-1.553) |
| Lag 2 | 1.021(0.938-1.111) | 1.059(0.920-1.219) | 1.100(0.892-1.356) | 1.182(0.817-1.711) |
| Lag 3 | 1.051(0.988-1.117) | 1.102(1.003-1.212)^*^ | 1.148(1.001-1.317)^*^ | 1.232(0.966-1.570) |
| Lag 4 | 1.060(0.988-1.137) | 1.097(0.977-1.231) | 1.119(0.941-1.330) | 1.150(0.844-1.565) |
| Lag 5 | 1.052(0.993-1.114) | 1.053(0.962-1.153) | 1.035(0.907-1.181) | 0.987(0.781-1.248) |
| Lag 6 | 1.033(0.975-1.093) | 0.987(0.906-1.076) | 0.924(0.818-1.044) | 0.801(0.646-0.993) |
| Lag 7 | 1.008(0.907-1.120) | 0.915(0.768-1.089) | 0.811(0.623-1.055) | 0.632(0.394-1.016) |
| **Cumulative lag effects RR (95% CI)** | | | | |
| Lag 0-1 | 1.009(0.841-1.210) | 0.969(0.744-1.262) | 0.919(0.645-1.308) | 0.823(0.457-1.485) |
| Lag 0-2 | 1.030(0.838-1.265) | 1.026(0.768-1.371) | 1.011(0.695-1.471) | 0.974(0.526-1.803) |
| Lag 0-3 | 1.082(0.867-1.350) | 1.131(0.838-1.528) | 1.160(0.798-1.688) | 1.199(0.658-2.184) |
| Lag 0-4 | 1.147(0.903-1.457) | 1.241(0.899-1.713) | 1.299(0.872-1.933) | 1.379(0.732-2.596) |
| Lag 0-5 | 1.207(0.930-1.566) | 1.307(0.918-1.860) | 1.344(0.865-2.087) | 1.361(0.671-2.758) |
| Lag 0-6 | 1.246(0.948-1.637) | 1.290(0.898-1.854) | 1.242(0.799-1.929) | 1.090(0.544-2.186) |
| Lag 0-7 | 1.256(0.935-1.686) | 1.180(0.801-1.737) | 1.007(0.632-1.603) | 0.690(0.331-1.437) |

*: P＜0.05

**Table S5.** Relative risks (RRs) of allergic conjunctivitis outpatient visits for SO_2_ (75th, 90th, 95th and 99th percentiles) exposure at various lag days, with the reference value of 25th percentile.

| Lag effects | 75th percentile | 90th percentile | 95th percentile | 99th percentile |
| --- | --- | --- | --- | --- |
| **Single lag effects RR (95% CI)** | | | | |
| Lag 0 | 0.980(0.867-1.107) | 0.929(0.676-1.276) | 0.866(0.552-1.358) | 0.563(0.226-1.401) |
| Lag 1 | 1.005(0.934-1.081) | 1.010(0.834-1.222) | 1.008(0.769-1.321) | 0.953(0.566-1.606) |
| Lag 2 | 1.007(0.944-1.073) | 1.021(0.864-1.207) | 1.036(0.817-1.315) | 1.113(0.710-1.746) |
| Lag 3 | 0.998(0.953-1.044) | 0.997(0.884-1.124) | 1.002(0.844-1.188) | 1.066(0.773-1.469) |
| Lag 4 | 0.990(0.938-1.044) | 0.975(0.848-1.121) | 0.969(0.794-1.183) | 1.003(0.687-1.463) |
| Lag 5 | 0.983(0.943-1.026) | 0.957(0.857-1.069) | 0.941(0.804-1.102) | 0.939(0.700-1.262) |
| Lag 6 | 0.978(0.939-1.018) | 0.942(0.847-1.047) | 0.917(0.789-1.067) | 0.878(0.666-1.158) |
| Lag 7 | 0.973(0.899-1.053) | 0.928(0.754-1.143) | 0.895(0.665-1.205) | 0.819(0.469-1.430) |
| **Cumulative lag effects RR (95% CI)** | | | | |
| Lag 0-1 | 0.984(0.865-1.120) | 0.938(0.672-1.309) | 0.873(0.547-1.392) | 0.536(0.203-1.419) |
| Lag 0-2 | 0.991(0.861-1.141) | 0.958(0.667-1.376) | 0.904(0.545-1.502) | 0.597(0.214-1.667) |
| Lag 0-3 | 0.989(0.854-1.144) | 0.955(0.655-1.391) | 0.906(0.535-1.533) | 0.636(0.227-1.780) |
| Lag 0-4 | 0.978(0.837-1.143) | 0.931(0.623-1.391) | 0.878(0.501-1.539) | 0.638(0.220-1.851) |
| Lag 0-5 | 0.962(0.813-1.138) | 0.891(0.578-1.374) | 0.826(0.451-1.514) | 0.599(0.193-1.860) |
| Lag 0-6 | 0.941(0.794-1.114) | 0.839(0.542-1.299) | 0.758(0.413-1.393) | 0.526(0.169-1.637) |
| Lag 0-7 | 0.915(0.767-1.092) | 0.779(0.495-1.225) | 0.678(0.362-1.272) | 0.431(0.132-1.410) |

**Table S6.** Relative risks (RRs) of allergic conjunctivitis outpatient visits for O_3_ (75th, 90th, 95th and 99th percentiles) exposure at various lag days, with the reference value of 25th percentile.

| Lag effects | 75th percentile | 90th percentile | 95th percentile | 99th percentile |
| --- | --- | --- | --- | --- |
| **Single lag effects RR (95% CI)** | | | | |
| Lag 0 | 0.907(0.662-1.241) | 0.830(0.585-1.180) | 0.799(0.554-1.153) | 0.732(0.483-1.108) |
| Lag 1 | 0.879(0.712-1.084) | 0.867(0.689-1.090) | 0.866(0.683-1.096) | 0.867(0.668-1.126) |
| Lag 2 | 0.915(0.768-1.091) | 0.915(0.756-1.108) | 0.918(0.754-1.119) | 0.929(0.746-1.156) |
| Lag 3 | 0.975(0.850-1.119) | 0.963(0.830-1.117) | 0.958(0.823-1.115) | 0.948(0.803-1.118) |
| Lag 4 | 1.015(0.866-1.190) | 0.999(0.842-1.184) | 0.990(0.832-1.178) | 0.969(0.803-1.170) |
| Lag 5 | 1.036(0.914-1.175) | 1.023(0.893-1.173) | 1.016(0.883-1.168) | 0.996(0.855-1.160) |
| Lag 6 | 1.044(0.923-1.180) | 1.040(0.910-1.189) | 1.037(0.904-1.190) | 1.027(0.883-1.194) |
| Lag 7 | 1.044(0.825-1.321) | 1.053(0.819-1.356) | 1.056(0.817-1.366) | 1.061(0.803-1.401) |
| **Cumulative lag effects RR (95% CI)** | | | | |
| Lag 0-1 | 0.796(0.555-1.142) | 0.720(0.479-1.080) | 0.692(0.452-1.059) | 0.634(0.390-1.032) |
| Lag 0-2 | 0.729(0.484-1.098) | 0.659(0.416-1.044) | 0.635(0.392-1.030) | 0.589(0.339-1.024) |
| Lag 0-3 | 0.711(0.468-1.081) | 0.634(0.394-1.021) | 0.609(0.369-1.005) | 0.558(0.312-0.998) |
| Lag 0-4 | 0.722(0.467-1.115) | 0.633(0.385-1.042) | 0.603(0.356-1.020) | 0.541(0.293-0.999) |
| Lag 0-5 | 0.748(0.474-1.179) | 0.648(0.384-1.095) | 0.612(0.351-1.067) | 0.539(0.281-1.033) |
| Lag 0-6 | 0.781(0.501-1.215) | 0.674(0.401-1.135) | 0.635(0.364-1.108) | 0.554(0.286-1.074) |
| Lag 0-7 | 0.815(0.521-1.275) | 0.710(0.415-1.215) | 0.671(0.376-1.196) | 0.588(0.293-1.178) |

**Table S7.** Correlation of air pollutants exposure (75th, 90th, 95th and 99th percentiles), and outpatients for allergic conjunctivitis：Multi-pollutant model.

|  | PM_2.5_ | | PM_10_ | | CO | | NO_2_ | | SO_2_ | | O_3_ | |
| --- | --- | --- | --- | --- | --- | --- | --- | --- | --- | --- | --- | --- |
|  | Single lag effects | Cumulative lag effects | Single lag effects | Cumulative lag effects | Single lag effects | Cumulative lag effects | Single lag effects | Cumulative lag effects | Single lag effects | Cumulative lag effects | Single lag effects | Cumulative lag effects |
| Adjusted for PM_2.5_ | - | - | 1.066(1.002-1.135)^*^  1.124(1.006-1.256)^*^  1.169(1.013-1.350)^*^  1.292(1.036-1.611)^*^ | 1.216(0.934-1.584)  1.385(0.889-2.158)  1.588(0.889-2.835)  2.051(0.867-4.852) | 1.169(0.921-1.485)  1.345(0.823-2.199)  1.399(0.753-2.599)  0.815(0.592-1.123) | 1.195(0.917-1.557)  1.432(0.840-2.441)  1.684(0.821-3.452)  2.791(0.884-8.812) | 1.052(0.993-1.115)  1.098(0.998-1.208)  1.142(0.995-1.310)  0.798(0.643-0.991)^*^ | 1.361(1.006-1.841)^*^  1.429(0.946-2.157)  1.422(0.830-2.437)  0.664(0.263-1.672) | 0.976(0.937-1.017)  0.938(0.843-1.044)  0.911(0.782-1.061)  0.587(0.224-1.537) | 0.914(0.758-1.103)  0.774(0.477-1.255)  0.667(0.340-1.308)  0.391(0.112-1.359) | 0.883(0.715-1.091)  0.874(0.694-1.100)  0.801(0.554-1.158)  0.735(0.484-1.116) | 0.705(0.461-1.078)  0.631(0.390-1.020)  0.606(0.366-1.005)  0.530(0.286-0.983)^*^ |
| Adjusted for PM_10_ | 1.081(0.956-1.223)  1.166(0.929-1.463)  1.211(0.920-1.595)  1.248(0.856-1.821) | 1.201(0.872-1.654)  1.389(0.767-2.515)  1.468(0.696-3.096)  1.400(0.520-3.770) | - | - | 1.169(0.921-1.485)  1.345(0.823-2.199)  1.399(0.753-2.599)  0.815(0.592-1.123) | 1.195(0.919-1.478)  1.432(0.837-2.150)  1.684(0.831-2.951)  2.526(0.808-5.867) | 1.052(0.993-1.115)  1.098(0.998-1.208)  1.142(0.995-1.310)  0.798(0.643-0.991)^*^ | 1.298(0.967-1.741)  1.385(0.937-2.046)  1.433(0.876-2.344)  1.470(0.718-3.012) | 0.977(0.939-1.018)  0.940(0.846-1.046)  0.915(0.786-1.065)  0.582(0.229-1.478) | 0.922(0.767-1.109)  0.794(0.495-1.275)  0.696(0.361-1.342)  0.438(0.131-1.469) | 0.879(0.712-1.086)  0.870(0.691-1.095)  0.791(0.547-1.143)  0.724(0.477-1.098) | 0.705(0.463-1.073)  0.632(0.392-1.019)  0.608(0.368-1.006)  0.561(0.313-1.006) |
| Adjusted for CO | 1.096(0.972-1.234)  1.197(0.961-1.491)  1.254(0.961-1.636)  0.481(0.210-1.101) | 0.865(0.667-1.121)  0.722(0.444-1.173)  0.623(0.343-1.134)  0.481(0.210-1.101) | 1.078(1.014-1.146)^*^  1.147(1.029-1.278)^*^  1.200(1.042-1.382)^**^  1.346(1.083-1.672)^*^ | 0.883(0.765-1.020)  0.798(0.621-1.026)  0.742(0.538-1.024)  0.621(0.377-1.023) | - | - | 1.051(0.992-1.113)  1.107(1.006-1.217)^*^  1.152(1.005-1.322)^*^  0.800(0.645-0.992)^*^ | 1.185(0.888-1.580)  0.861(0.624-1.188)  0.774(0.495-1.210)  0.535(0.224-1.277) | 0.976(0.937-1.017)  0.938(0.843-1.044)  0.912(0.783-1.062)  0.496(0.196-1.255) | 0.878(0.727-1.062)  0.701(0.431-1.141)  0.588(0.299-1.157)  0.381(0.113-1.286) | 0.883(0.715-1.090)  0.870(0.692-1.095)  0.803(0.556-1.159)  0.731(0.483-1.108) | 0.726(0.476-1.105)  0.645(0.4-1.039)  0.617(0.373-1.020)  0.561(0.314-1.005) |
| Adjusted for NO_2_ | 1.091(0.968-1.229)  1.185(0.952-1.476)  1.236(0.948-1.613)  1.275(0.882-1.843) | 1.157(0.876-1.528)  1.293(0.780-2.144)  1.332(0.709-2.505)  0.697(0.312-1.557) | 1.081(1.016-1.150)^*^  1.151(1.032-1.285)^*^  1.206(1.046-1.391)^**^  1.354(1.088-1.686)^**^ | 1.149(0.930-1.420)  1.287(0.896-1.848)  1.398(0.886-2.206)  1.715(0.875-3.361) | 1.153(0.909-1.462)  1.287(0.793-2.090)  0.712(1.305-0.378)  0.814(0.591-1.121) | 1.190(0.924-1.532)  1.492(0.818-2.720)  1.605(0.846-3.047)  2.532(0.865-7.412) | - | - | 0.976(0.938-1.017)  0.938(0.843-1.044)  0.912(0.783-1.062)  0.521(0.197-1.377) | 0.900(0.739-1.097)  0.743(0.446-1.240)  0.632(0.309-1.294)  0.377(0.102-1.39) | 0.883(0.715-1.091)  0.830(0.584-1.180)  0.798(0.552-1.152)  0.726(0.479-1.101) | 0.718(0.472-1.094)  0.644(0.399-1.040)  0.620(0.374-1.026)  0.550(0.297-1.018) |
| Adjusted for SO_2_ | 1.091(0.967-1.231)  1.189(0.953-1.484)  1.248(0.954-1.631)  1.308(0.902-1.898) | 1.161(0.897-1.502)  1.324(0.837-2.095)  1.393(0.815-2.382)  1.439(0.670-3.089) | 1.081(1.016-1.150)^*^  1.152(1.033-1.285)^**^  1.208(1.048-1.391)^**^  1.358(1.092-1.688)^**^ | 1.156(0.944-1.416)  1.300(0.921-1.834)  1.413(0.918-2.174)  1.723(0.915-3.244) | 1.115(0.896-1.389)  1.199(0.773-1.861)  1.120(0.796-1.577)  0.805(0.585-1.107) | 1.193(0.883-1.613)  1.390(0.800-2.413)  1.486(0.839-2.632)  2.431(0.916-6.448) | 1.051(0.993-1.114)  1.102(1.002-1.212)^*^  1.147(0.999-1.317)  0.800(0.645-0.992) | 1.254(0.943-1.666)  1.368(0.934-2.003)  1.474(0.908-2.392)  1.662(0.820-3.372) | - | - | 0.865(0.700-1.070)  0.854(0.678-1.076)  0.854(0.673-1.083)  0.721(0.474-1.095) | 0.685(0.449-1.046)  0.611(0.378-0.989)^*^  0.588(0.354-0.975)^*^  0.541(0.301-0.973)^*^ |
| Adjusted for O_3_ | 1.098(0.975-1.238)  1.203(0.965-1.498)  1.261(0.966-0.647)  0.575(0.287-1.152) | 0.889(0.710-1.114)  0.773(0.510-1.171)  0.693(0.418-1.149)  0.575(0.287-1.152) | 1.079(1.015-1.147)^*^  1.149(1.031-1.281)^**^  1.203(1.045-1.385)^**^  1.351(1.088-1.678)^**^ | 0.889(0.779-1.015)  0.808(0.643-1.014)  0.753(0.563-1.006)  0.630(0.401-0.991)^*^ | 1.022(0.945-1.105)  1.018(0.882-1.174)  0.608(0.862-1.287)  1.403(0.834-2.361) | 1.118(0.833-1.501)  1.213(0.711-2.070)  1.245(0.705-0.441)  1.761(0.700-4.427) | 1.055(0.996-1.118)  1.112(1.011-1.223)^*^  1.161(1.012-1.333)^*^  0.805(0.649-0.999)^*^ | 1.278(0.972-1.680)  1.330(0.934-1.894)  1.347(0.866-2.095)  0.674(0.321-1.415) | 0.983(0.943-1.023)  0.953(0.856-1.060)  0.930(0.799-1.083)  0.520(0.207-1.306) | 0.934(0.781-1.117)  0.816(0.516-1.290)  0.714(0.379-1.347)  0.402(0.122-1.321) | - | - |
| Adjusted for the other 5 pollutants | 1.092(0.963-1.237)  1.195(0.949-1.504)  1.262(0.954-1.668)  1.345(0.917-1.973) | 1.178(0.800-1.733)  1.307(0.630-2.711)  0.765(0.318-1.845)  0.567(0.176-1.828) | 1.078(1.011-1.150)^*^  1.147(1.024-1.285)^*^  1.199(1.035-1.389)^*^  1.340(1.070-1.677)^**^ | 1.260(0.959-1.655)  1.511(0.944-2.419)  1.714(0.944-3.112)  2.269(0.939-5.483) | 1.100(0.850-1.424)  1.188(0.697-2.022)  1.129(0.790-1.613)  0.812(0.589-1.120) | 1.188(0.830-1.701)  1.403(0.712-2.764)  1.562(0.727-3.353)  2.752(0.817-9.273) | 1.055(0.995-1.118)  1.115(1.012-1.229)^*^  1.166(1.041-1.339)^*^  0.808(0.65-1.004) | 1.398(1.016-1.924)^*^  1.527(0.974-2.394)  1.585(0.875-2.873)  1.608(0.649-3.988) | 0.978(0.938-1.019)  0.941(0.844-1.050)  0.916(0.781-1.069)  0.541(0.200-1.459) | 0.926(0.752-1.140)  0.799(0.467-1.367)  0.697(0.331-1.470)  0.411(0.109-1.553) | 0.886(0.714-1.100)  0.882(0.697-1.117)  0.803(0.551-1.171)  0.724(0.473-1.110) | 0.716(0.464-1.105)  0.625(0.374-1.043)  0.594(0.345-1.021)  0.531(0.282-0.999)^*^ |

*: P＜0.05; **: P＜0.01.

**Table S8.** Correlation of air pollutants exposure (75th, 90th, 95th and 99th percentiles), and outpatients for allergic conjunctivitis, and effect modification through stratified by characteristics of patients.

| Characteristics | PM_2.5_ | | PM_10_ | | CO | | NO_2_ | | SO_2_ | | O_3_ | |
| --- | --- | --- | --- | --- | --- | --- | --- | --- | --- | --- | --- | --- |
|  | Single lag effects | Cumulative lag effects | Single lag effects | Cumulative lag effects | Single lag effects | Cumulative lag effects | Single lag effects | Cumulative lag effects | Single lag effects | Cumulative lag effects | Single lag effects | Cumulative lag effects |
| **Sex** | | | | | | | | | | | | |
| Male | 1.118(0.927-1.348)  1.234(0.874-1.742)  1.282  (0.837-1.965)  0.784(0.569-1.080) | 1.183(0.709-1.974)  1.359(0.516-3.577)  1.413(0.417-4.786)  1.493(0.264-8.455) | 1.058(0.967-1.158)  1.107(0.946-1.295)  1.144(0.936-1.398)  1.237(0.906-1.688) | 0.925(0.688-1.243)  0.851(0.516-1.406)  0.789(0.419-1.487)  0.601(0.213-1.694) | 0.957(0.886-1.034)  1.349(0.738-2.464)  1.441(0.646-3.216)  0.703(0.448-1.102) | 1.145(0.89-1.474)  1.411(0.734-2.713)  1.532(0.652-3.597)  3.044(0.628-14.754) | 1.038(0.960-1.121)  1.064(0.932-1.215)  0.869(0.726-1.039)  0.697(0.503-0.966) | 1.364(0.863-2.156)  1.304(0.731-2.325)  1.313(0.597-2.892)  0.421(0.091-1.941) | 0.968(0.912-1.027)  0.921(0.789-1.075)  0.895(0.718-1.117)  0.125(0.009-1.680) | 1.066(0.845-1.344)  0.778(0.365-1.662)  0.556(0.195-1.587)  0.050(0.002-1.068) | 0.806(0.529-1.228)  0.737(0.462-1.177)  0.713(0.438-1.159)  0.662(0.383-1.143) | 0.645(0.363-1.145)  0.529(0.270-1.039)  0.488(0.24-0.991)  0.380(0.159-0.906) |
| Female | 1.083(0.983-1.193)  1.165(0.977-1.388)  1.204(0.977-1.483)  1.375(0.893-2.117) | 1.248(0.788-1.977)  1.430(0.603-3.393)  0.697(0.255-1.903)  0.485(0.130-1.812) | 1.095(1.018-1.178)^*^  1.179(1.036-1.342)^*^  1.244(1.052-1.471)^**^  1.416(1.096-1.829)^**^ | 3.999(1.475-10.844)^**^  12.869(2.174-76.179)^**^  2.279(1.195-4.343)^*^  3.308(1.349-8.112)^**^ | 1.026(0.960-1.098)  1.078(0.919-1.265)  1.167(0.869-1.568)  1.561(0.870-2.802) | 1.181(0.903-1.545)  1.516(0.799-2.875)  1.677(0.771-3.649)  2.474(0.677-9.046) | 1.088(1.002-1.182)^*^  1.149(1.029-1.283)^*^  1.214(1.041-1.416)^*^  1.335(1.026-1.738)^*^ | 1.453(1.010-2.091)^*^  1.667(1.003-2.771)^*^  1.803(0.933-3.485)  1.947(0.678-5.590) | 0.976(0.930-1.024)  0.935(0.825-1.060)  0.905(0.758-1.080)  0.835(0.608-1.148) | 0.941(0.746-1.186)  0.843(0.463-1.536)  0.77(0.334-1.778)  0.630(0.228-1.743) | 0.893(0.725-1.099)  0.894(0.679-1.176)  0.899(0.676-1.194)  0.776(0.469-1.286) | 0.775(0.470-1.277)  0.722(0.408-1.278)  0.705(0.386-1.289)  0.711(0.393-1.284) |
| **Age(years)** | | | | | | | | | | | | |
| 0-1 | 2.030(1.117-3.691)*  3.297(1.039-10.467)*  4.569(1.35-15.462)*  27.203(3.632-203.752)** | 27.174(3.330-221.759)**  193.952  (1.607-23409.890)*  95.959(0.018-510508.625)  0.000(0.000-508.526) | 1.333(1.105-1.608)**  1.691(1.201-2.379)**  2.026(1.295-3.170)**  3.282(1.615-6.668)** | 3.999(1.475-10.844)**  12.869(2.174-76.179)**  31.029（3.041-316.629）**  324.030  (8.504-12347.276)** | 0.238(0.079-0.713)**  8.391(2.261-31.136)**  45.855(4.063-517.528)**  0.000(0.000-0.000)** | 0.238(0.079-0.713)**  0.00(0.00-0.031)**  0.000(0.000-0.001)**  0.000(0.000-0.000)** | 1.446(1.117-1.871)**  1.753(1.139-2.700)**  0.000 （0.000-0.269）**  0.000(0.000-0.089)* | 0.493(0.192-1.267)  0.018(0.001-0.331)**  0.001(0.000-0.096)**  0.000(0.000-0.011)** | 1.196(0.905-1.581)  2.45(1.349-4.452)**  10.798(2.256-51.695)**  99845868850.829(735.346-13557144385785092096) | 0.697(0.271-1.791)  20.303(1.329-310.089)*  7334.762(37.630-1429678.513)**  5.15926874480295e+27(38803860231.481-6.85964072190581e+44)** | 2.552(1.461-4.459)**  2.586(1.474-4.537)**  2.501(1.417-4.415)**  3.157(1.557-6.400)** | 93.389(6.284-1387.928)**  131.584  (7.162-2417.370)**  130.114(6.7-2526.946)**  12.747(2.375-68.401)** |
| 2-5 | 1.198(0.911-1.577)  1.332(0.820-2.164)  0.806(0.568-1.145)  0.727(0.422-1.254) | 1.702(0.686-4.219)  2.705(0.511-14.310)  3.222(0.39-26.621)  3.034(0.097-95.170) | 1.074(0.943-1.224)  1.164(0.864-1.569)  0.499  （0.122-1.656）  0.132(0.011-1.594) | 0.804(0.448-1.440)  0.605(0.222-1.650)  0.449（0.122-1.656）  0.132(0.011-1.594) | 1.073(0.926-1.243)  1.073(0.926-1.243)  0.487(0.216-1.098)  0.124(0.011-1.400) | 1.567(0.924-2.658)  3.064(0.837-11.226)  3.912(0.713-21.448)  0.180(0.001-24.354) | 1.102(0.950-1.278)  0.423(0.164-1.092)  0.202(0.044-0.927)*  0.047(0.003-0.754)* | 0.778(0.438-1.384)  0.423(0.164-1.092)  0.126(0.020-0.809)*  0.011(0.000-0.313)** | 1.099(0.885-1.365)  0.884(0.653-1.196)  0.781(0.503-1.213)  0.167(0.011-2.637) | 1.188(0.749-1.885)  1.407(0.445-4.454)  0.462(0.055-3.866)  0.00(0.00-5.461) | 0.592(0.435-0.806)**  0.580(0.416-0.809)**  0.586(0.418-0.823)**  0.614(0.426-0.884)** | 0.187(0.065-0.539)**  0.152(0.049-0.474)**  0.130(0.039-0.429)**  0.093(0.024-0.37)** |
| 6-18 | 1.118(0.864-1.447)  1.216(0.826-1.789)  1.333(0.833-2.135)  0.688(0.432-1.095) | 0.789(0.428-1.453)  0.624(0.191-2.038)  0.550(0.124-2.447)  0.491(0.069-3.467) | 1.138(0.992-1.305)  1.236(0.981-1.556)  1.286(0.963-1.716)  1.179(0.931-1.493) | 1.888(1.171-3.042)**  2.902(1.314-6.407)**  3.668(1.381-9.742)**  4.317(0.947-19.671) | 1.129(0.849-1.503)  0.895(0.727-1.101)  0.800(0.614-1.041)  0.441(0.220-0.887)* | 1.129(0.849-1.503)  1.274(0.597-2.718)  0.649(0.205-2.052)  3.348(0.540-20.742) | 1.102(0.998-1.217)  1.358(0.762-2.421)  0.834(0.652-1.068)  0.578(0.367-0.912)* | 1.530(0.865-2.706)  1.358(0.762-2.421)  1.770(0.692-4.529)  2.662(0.582-12.186) | 0.947(0.876-1.023)  0.864(0.704-1.059)  0.809(0.603-1.084)  0.344(0.071-1.677) | 0.745(0.537-1.032)  0.447(0.190-1.051)  0.302(0.09-1.016)  0.136(0.020-0.934)* | 0.745(0.520-1.067)  0.747(0.506-1.103)  0.757(0.507-1.129)  0.791(0.511-1.223) | 0.507(0.245-1.046)  0.444(0.198-0.999)*  0.429(0.184-0.998)*  0.37(0.134-1.022)* |
| 19-64 | 0.953(0.872-1.041)  0.923(0.788-1.082)  1.195(0.858-1.665)  1.264(0.802-1.991) | 1.272(0.806-2.008)  1.464(0.618-3.465)  1.394(0.478-4.066)  0.609(0.153-2.413) | 1.094(1.011-1.185)*  1.175(1.023-1.349)*  1.234(1.034-1.474)*  1.379(1.053-1.806)* | 1.183(0.902-1.552)  1.355(0.842-2.182)  1.494(0.809-2.756)  1.943(0.748-5.051) | 1.059(0.937-1.196)  1.146(0.858-1.530)  1.240(0.860-1.786)  1.731(0.823-3.642) | 1.154(0.877-1.518)  1.492(0.773-2.881)  1.711(0.768-3.814)  2.997(0.769-11.688) | 1.068(0.992-1.150)  1.122(1.002-1.256)*  1.162(0.99-1.365)  1.229(0.929-1.626) | 1.373(0.958-1.968)  1.584(0.939-2.672)  1.634(0.861-3.104)  1.762(0.626-4.959) | 0.974(0.925-1.025)  0.926(0.812-1.057)  0.885(0.735-1.065)  0.746(0.501-1.110) | 1.030(0.843-1.258)  0.836(0.440-1.590)  0.724(0.299-1.750)  0.331(0.063-1.737) | 1.050(0.900-1.226)  0.877(0.568-1.355)  0.853(0.541-1.344)  0.798(0.476-1.339) | 0.839(0.506-1.391)  0.820(0.496-1.357)  0.805(0.474-1.365)  0.798(0.476-1.339) |
| ≥65 | 0.661(0.218-2.000)  0.661(0.218-2.000)  1.336(0.836-2.134)  1.856(1.074-3.207)** | 0.591(0.176-1.985)  0.34(0.034-3.369)  0.241(0.014-4.023)  0.157(0.005-5.458) | 1.188(1.027-1.374)*  1.322(1.041-1.679)*  1.387(1.034-1.860)*  1.485(0.821-2.685) | 3.445(1.312-9.048)*  6.515(1.419-29.925)*  7.473(1.205-46.344)*  2.406(0.100-57.997) | 1.153(0.995-1.336)  1.536(1.093-2.158)*  1.831(1.219-2.751)**  3.239(1.469-7.142)** | 2.997(0.769-11.688)  0.382(0.083-1.752)  0.221(0.033-1.485)  0.023(0.000-1.113) | 1.103(0.996-1.220)  1.290(1.064-1.564)**  1.479(1.122-1.948)**  1.884(1.173-3.026)** | 1.551(0.902-2.665)  2.294(0.839-6.275)  2.385(0.585-9.723)  0.272(0.026-2.898) | 1.219(1.053-1.411)**  1.686(1.154-2.464)**  2.097(1.234-3.564)**  2.658(1.139-6.203)* | 1.789(0.986-3.245)  4.503(0.969-20.930)*  7.959(0.952-66.528)  8.672(0.410-183.211) | 0.716(0.478-1.072)  0.653(0.417-1.022)  0.321(0.087-1.181)  0.242(0.054-1.083) | 0.326(0.077-1.386)  0.364(0.106-1.255)  0.242(0.034-1.71)  0.242(0.054-1.083) |
| **Season** | | | | | | | | | | | | |
| Warm  (April to September) | 1.050(0.965-1.144)  1.087(0.939-1.258)  1.103(0.923-1.317)  0.925(0.775-1.103) | 0.900(0.701-1.155)  0.838(0.535-1.313)  0.816(0.464-1.435)  0.832(0.369-1.875) | 1.113(1.027-1.206)**  1.113(1.027-1.206)**  1.219(1.051-1.414)**  1.226(0.991-1.517) | 1.242(0.881-1.751)  0.795(0.546-1.159)  0.752(0.478-1.184)  0.689(0.370-1.283) | 1.046(0.992-1.104)  1.060(0.979-1.147)  1.059(0.967-1.161)  0.869(0.672-1.125) | 1.087(0.851-1.388)  0.875(0.654-1.170)  0.826(0.587-1.164)  0.642(0.371-1.112) | 1.044(0.983-1.108)  1.100(0.965-1.255)  1.138(0.960-1.349)  1.219(0.895-1.661) | 1.476(1.009-2.158)**  1.759(1.079-2.866)**  1.994(1.077-3.693)*  2.069(0.964-4.441) | 1.023(0.968-1.081)  0.970(0.884-1.065)  0.966(0.844-1.105)  0.847(0.54-1.329) | 1.036(0.905-1.187)  1.053(0.753-1.474)  0.915(0.504-1.661)  0.626(0.225-1.741) | 0.826(0.684-0.996)*  0.742(0.580-0.949)*  0.689(0.516-0.920)*  0.591(0.401-0.872)** | 0.826(0.684-0.996)*  0.742(0.580-0.949)*  0.689(0.516-0.92)*  0.591(0.401-0.872)** |
| Cold  (October to March) | 0.858(0.765-0.961)**  0.809(0.698-0.937)**  0.799(0.683-0.936)**  0.663(0.420-1.046) | 1.518(0.761-3.031)  1.727(0.649-4.597)  1.721(0.562-5.273)  1.486(0.330-6.688) | 0.925(0.819-1.046)  0.925(0.819-1.046)  0.918(0.810-1.042)  0.885(0.744-1.053) | 0.805(0.511-1.267)  0.689(0.329-1.442)  0.628(0.259-1.523)  0.514(0.160-1.654) | 0.841(0.715-0.989)*  0.770(0.635-0.935)**  0.715(0.578-0.884)**  0.654(0.483-0.884)** | 0.344(0.137-0.862)*  0.271(0.089-0.827)*  0.283(0.087-0.921)*  0.383(0.090-1.635) | 0.780(0.646-0.941)**  0.780(0.646-0.941)**  0.591(0.422-0.827)**  0.699(0.546-0.895)** | 0.632(0.389-1.028)  0.468(0.219-1.001)*  0.415(0.176-0.975)*  0.321(0.097-1.066) | 0.808(0.677-0.963)*  0.664(0.473-0.934)*  0.599(0.383-0.935)*  1.308(0.869-1.969) | 0.757(0.464-1.237)  0.591(0.237-1.476)  0.524(0.168-1.632)  0.588(0.173-2.000) | 0.807(0.671-0.971)*  0.759(0.595-0.968)*  0.619(0.411-0.933)*  0.544(0.320-0.925)* | 0.342(0.179-0.653)**  0.200(0.078-0.513)**  0.180(0.055-0.590)**  0.195(0.042-0.916)* |

*: P＜0.05; **: P＜0.01

Figure S1. Concentration distribution over time for meteorological factors in Urumqi, Xinjiang during 2013-2020.


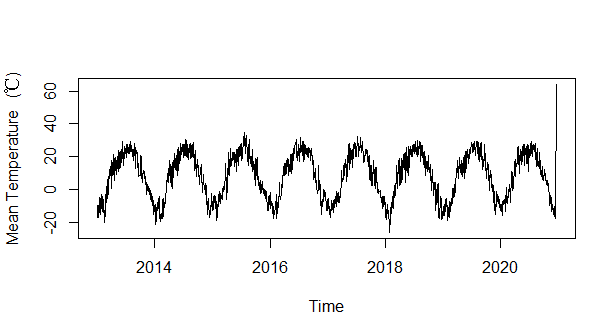

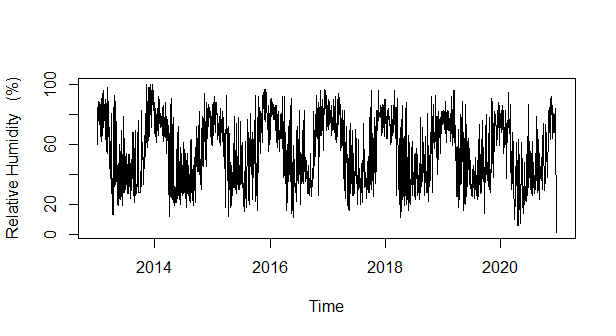

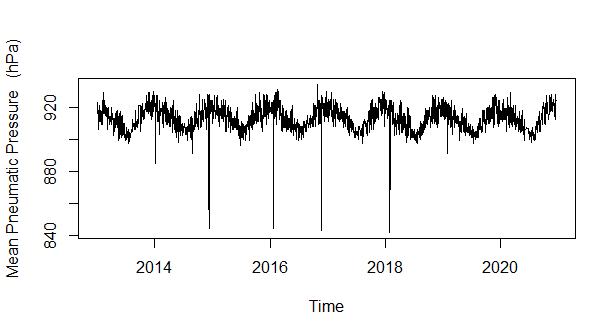


Figure S2. Exposure-response association between allergic conjunctivitis outpatient visits and PM_2.5_ exposure.


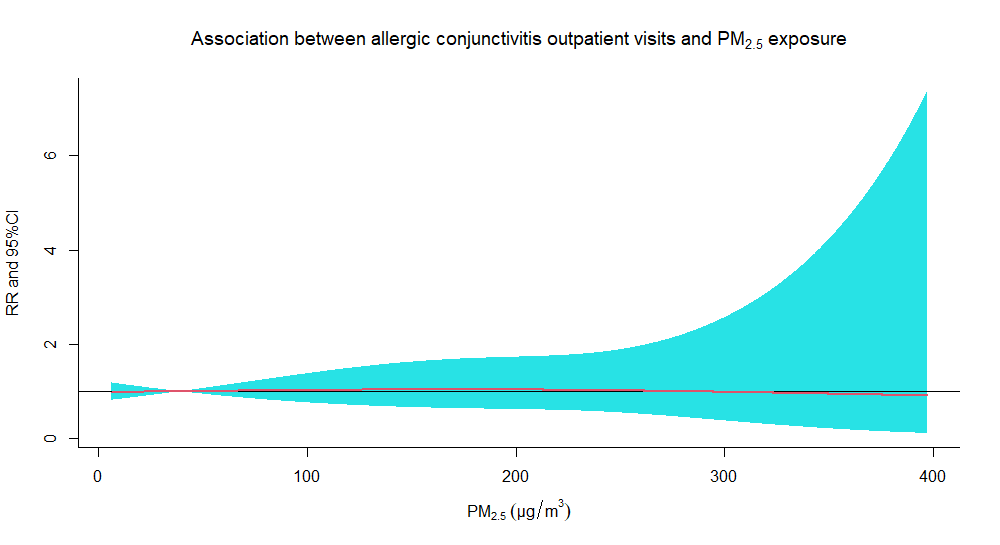


Figure S3. Exposure-response association between allergic conjunctivitis outpatient visits and PM_10_ exposure.


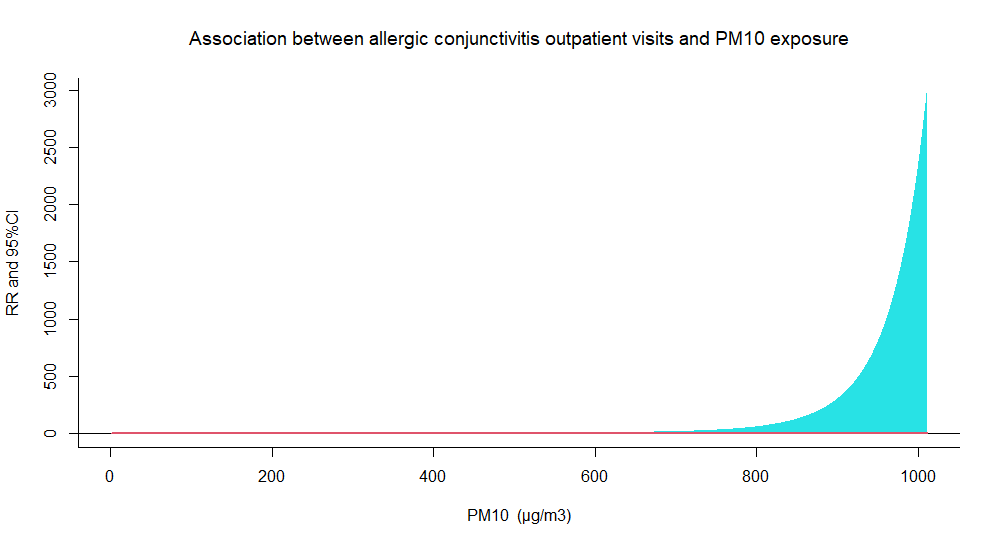


Figure S4. Exposure-response association between allergic conjunctivitis outpatient visits and CO exposure.


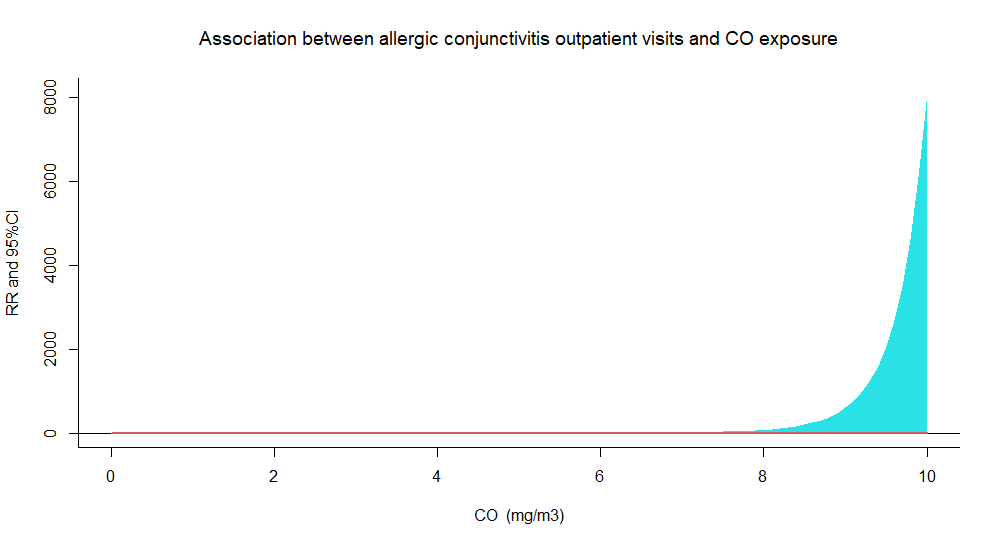


Figure S5. Exposure-response association between allergic conjunctivitis outpatient visits and NO_2_ exposure.


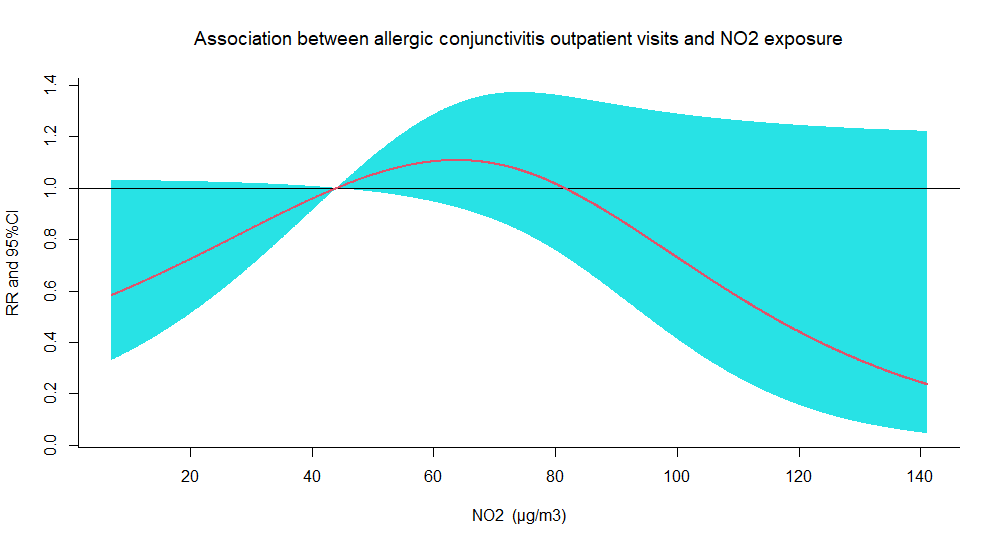


Figure S6. Exposure-response association between allergic conjunctivitis outpatient visits and SO_2_ exposure.


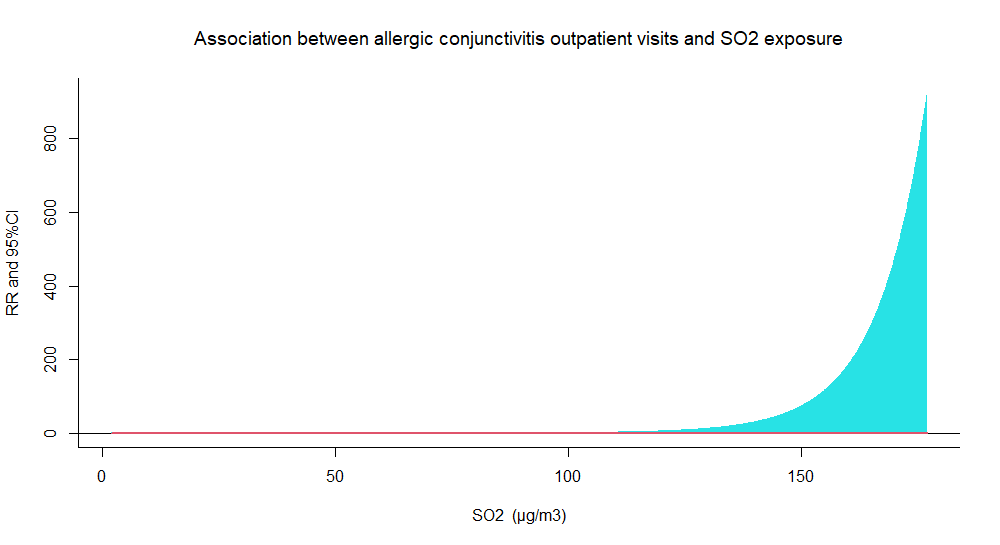


Figure S7. Exposure-response association between allergic conjunctivitis outpatient visits and O_3_ exposure.


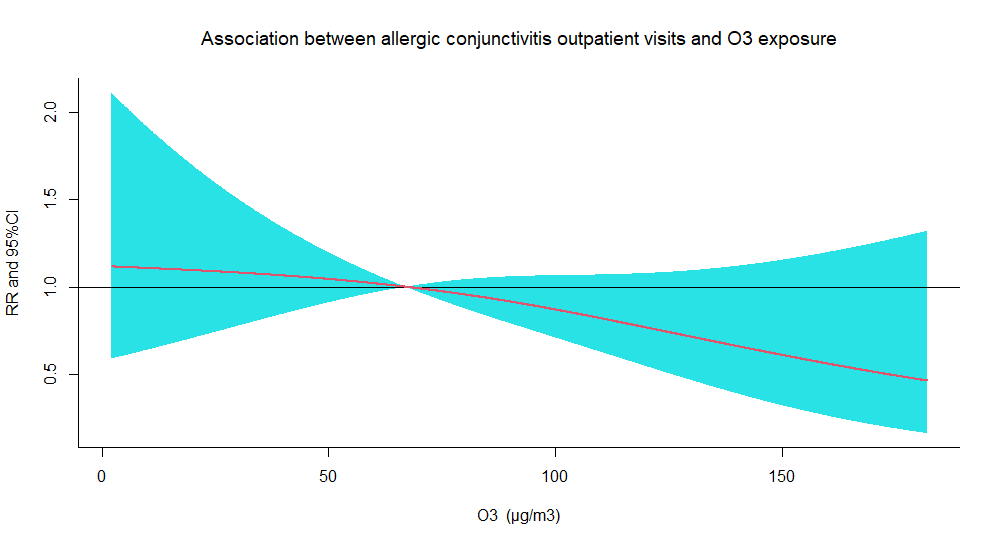


Figure S8. Overall exposure-response association and air pollution exposure distribution: Multipollutant model.


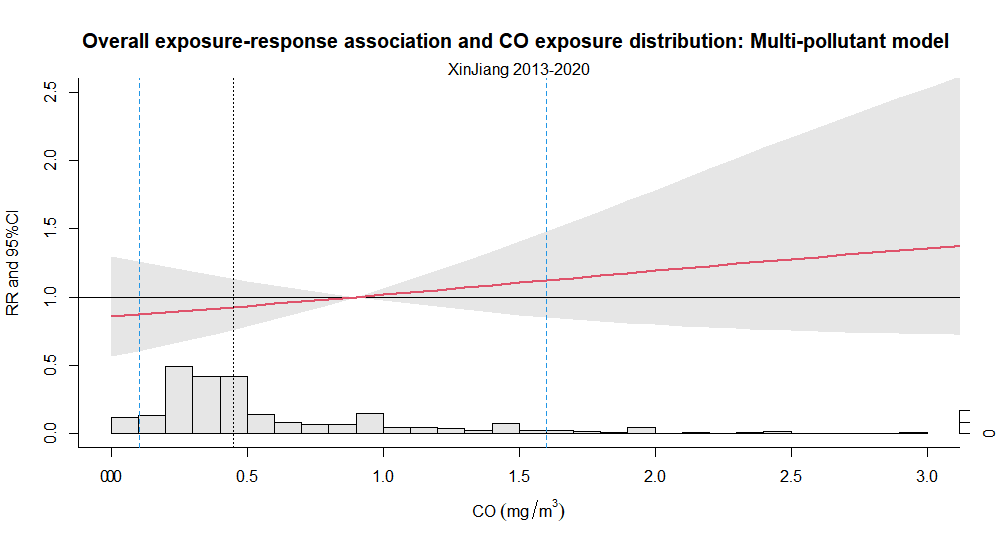

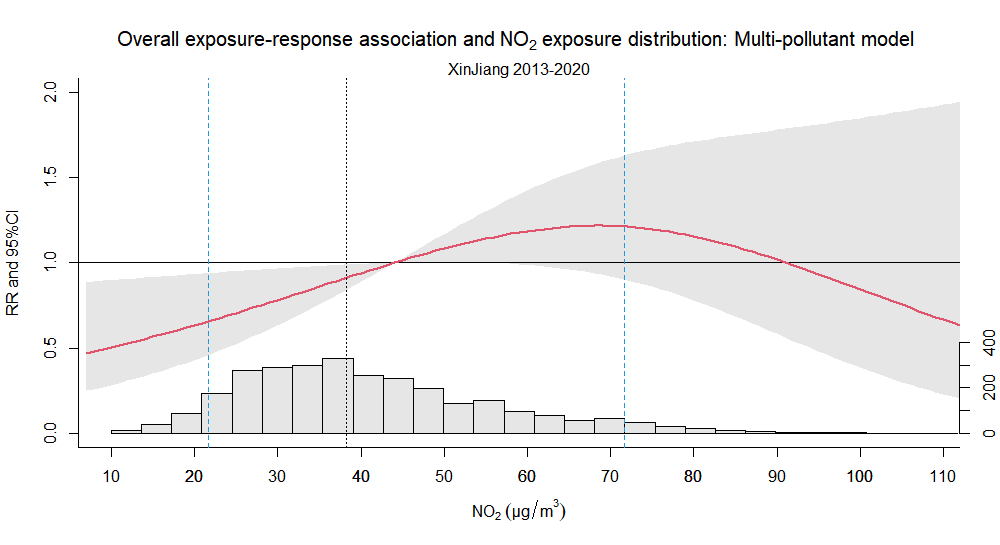

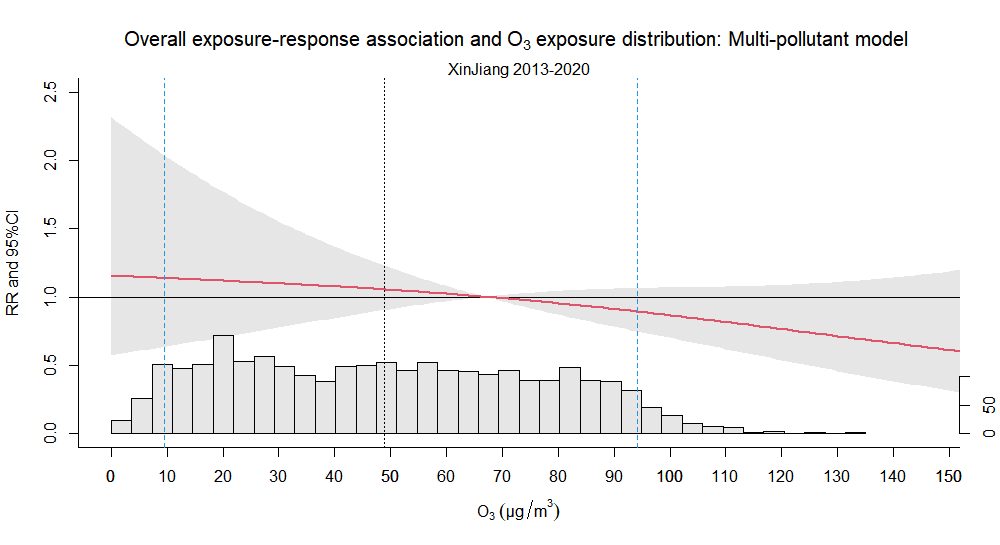

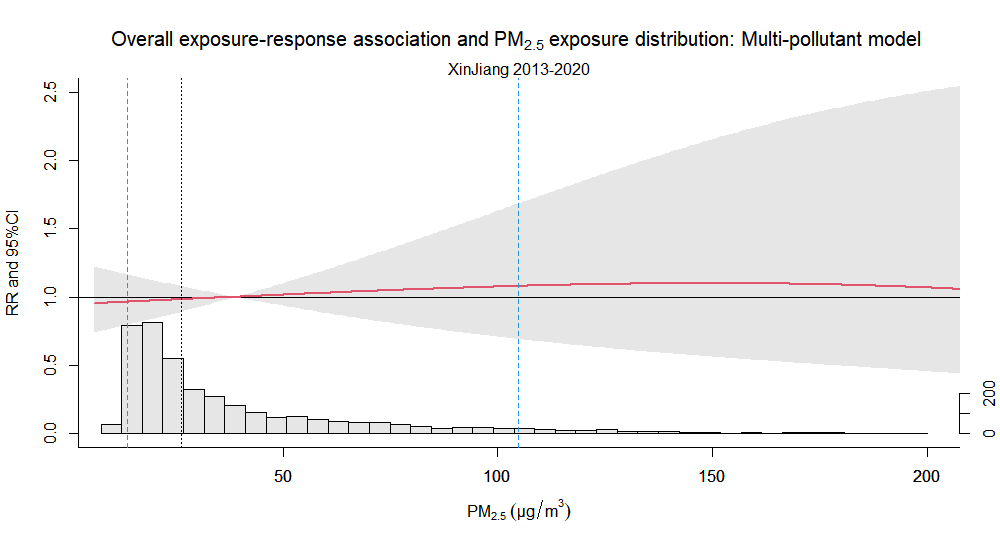

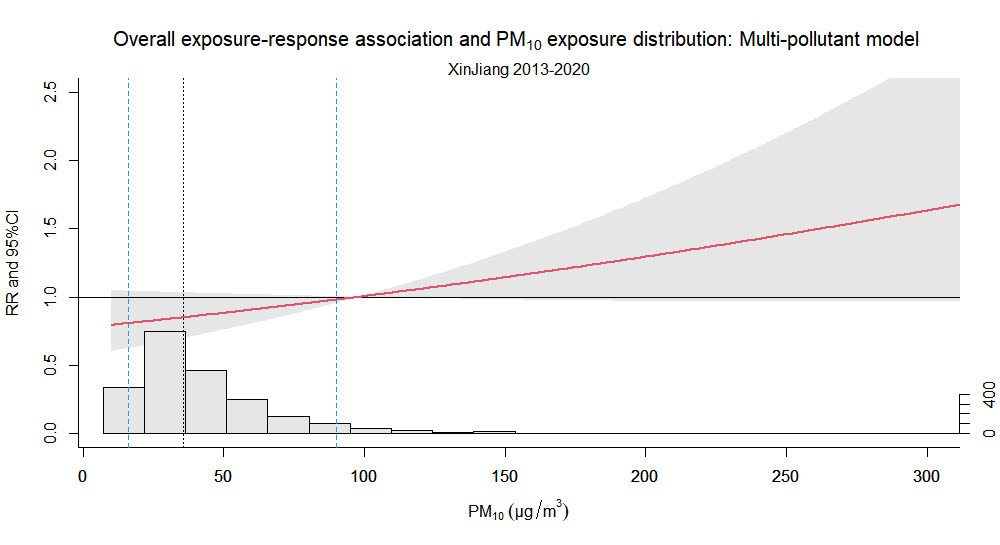

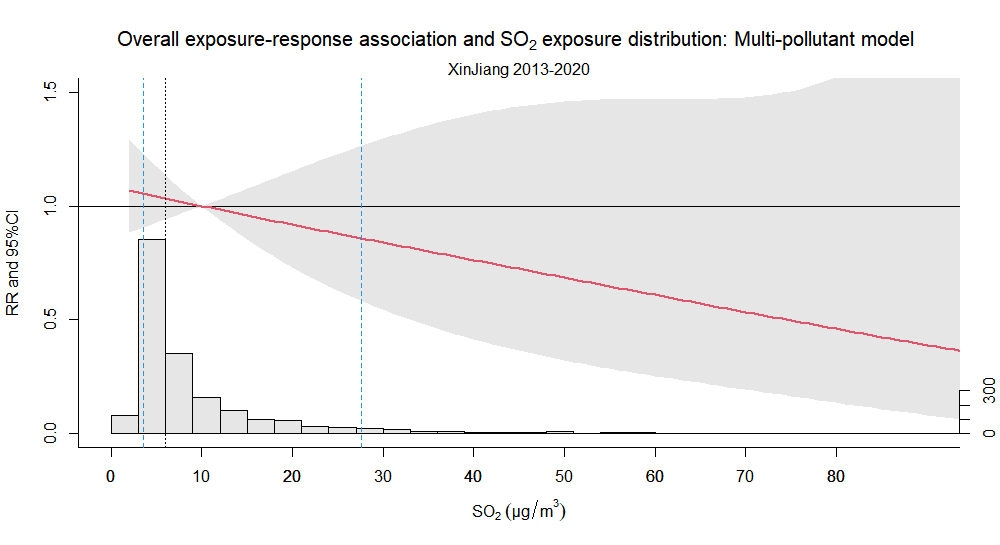

Supplement: Supplementary file 1 [file Data_Sheet_1.docx]
